# Supplementary material for: Comparative analysis reveals the long-term coevolutionary history of parvoviruses and vertebrates
Source: PLoS Biol. 2022 Nov 29;20(11):e3001867. doi: 10.1371/journal.pbio.3001867 (PMC9707805; doi:10.1371/journal.pbio.3001867)
Supplement: S1 Fig — (DOCX) [file pbio.3001867.s001.docx]

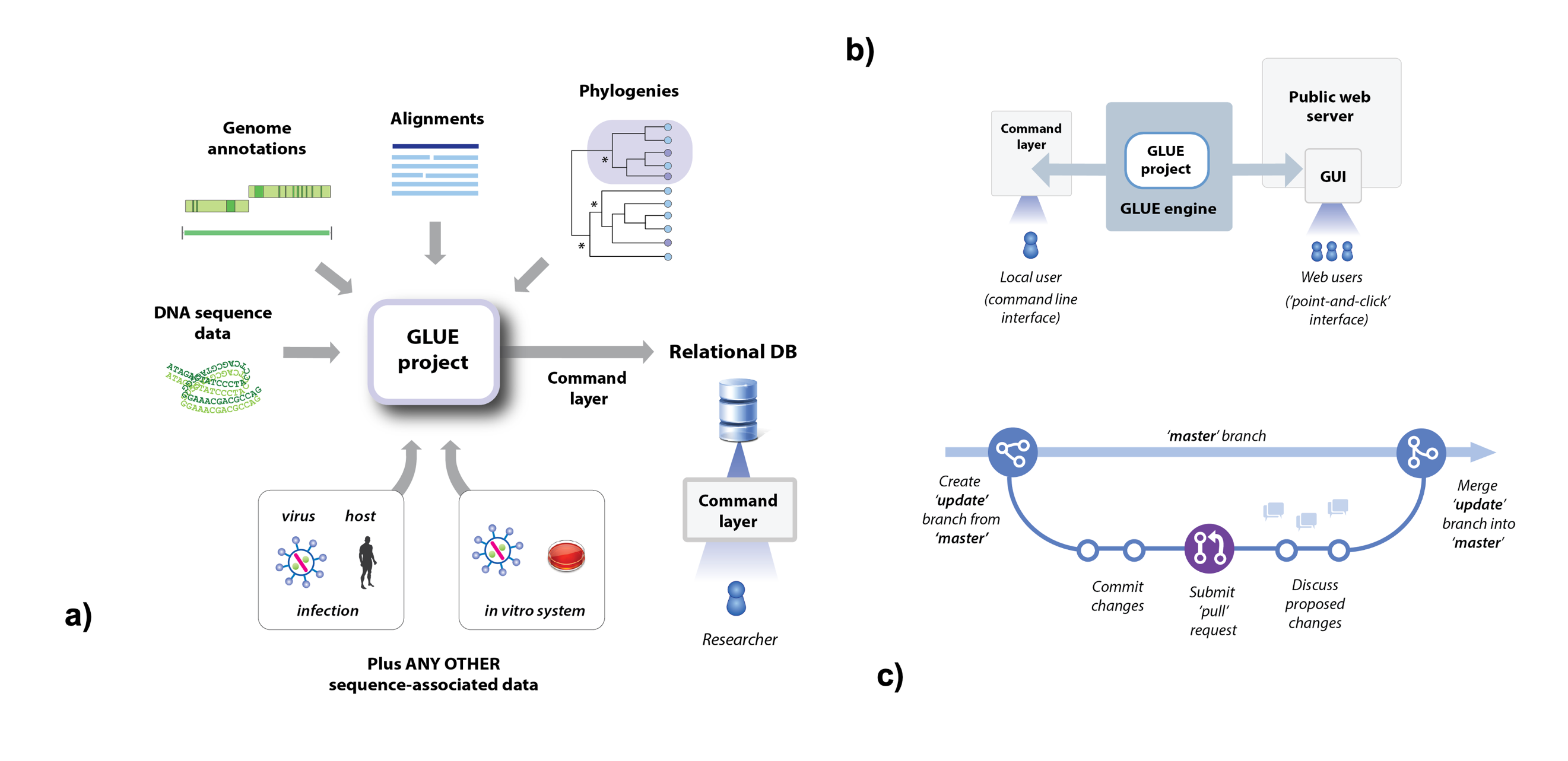


**Figure S1.** Parvovirus-GLUE – an open resource for reproducible comparative analysis of parvovirus genome data. **(a)** The GLUE software framework allows users to develop flexible ‘projects’ oriented around the data items involved in comparative sequence analysis. Typically, these will include molecular sequence data, genome annotations, multiple sequence alignments (MSAs) and phylogenies, as well as diverse other forms of data. Loading projects into the GLUE ‘engine’ creates a relational database that captures the semantic relationships between data items. The database is constructed using GLUE’s native command layer, which can also be used to interact with the database and with commonly used bioinformatics programs (e.g., BLAST, RAxML, MAAFT). Working within this framework makes analyses reproducible and enables re-use and development of complex data items. **(b)** Loading projects into the GLUE ‘engine’ creates a relational database that captures the semantic relationships between data items. The database is constructed using GLUE’s native command layer, which can also be used to develop analysis protocols that utilise data items by interacting with the database and with commonly used bioinformatics programs (e.g., BLAST, RAxML, MAAFT). **(c)** Hosting of GLUE projects in an online version control system (e.g., GitHub) allows controlled collaborative development.
