# Supplementary material for: Comparative analysis reveals the long-term coevolutionary history of parvoviruses and vertebrates
Source: PLoS Biol. 2022 Nov 29;20(11):e3001867. doi: 10.1371/journal.pbio.3001867 (PMC9707805; doi:10.1371/journal.pbio.3001867)
Supplement: S2 Fig — (DOCX) [file pbio.3001867.s002.docx]

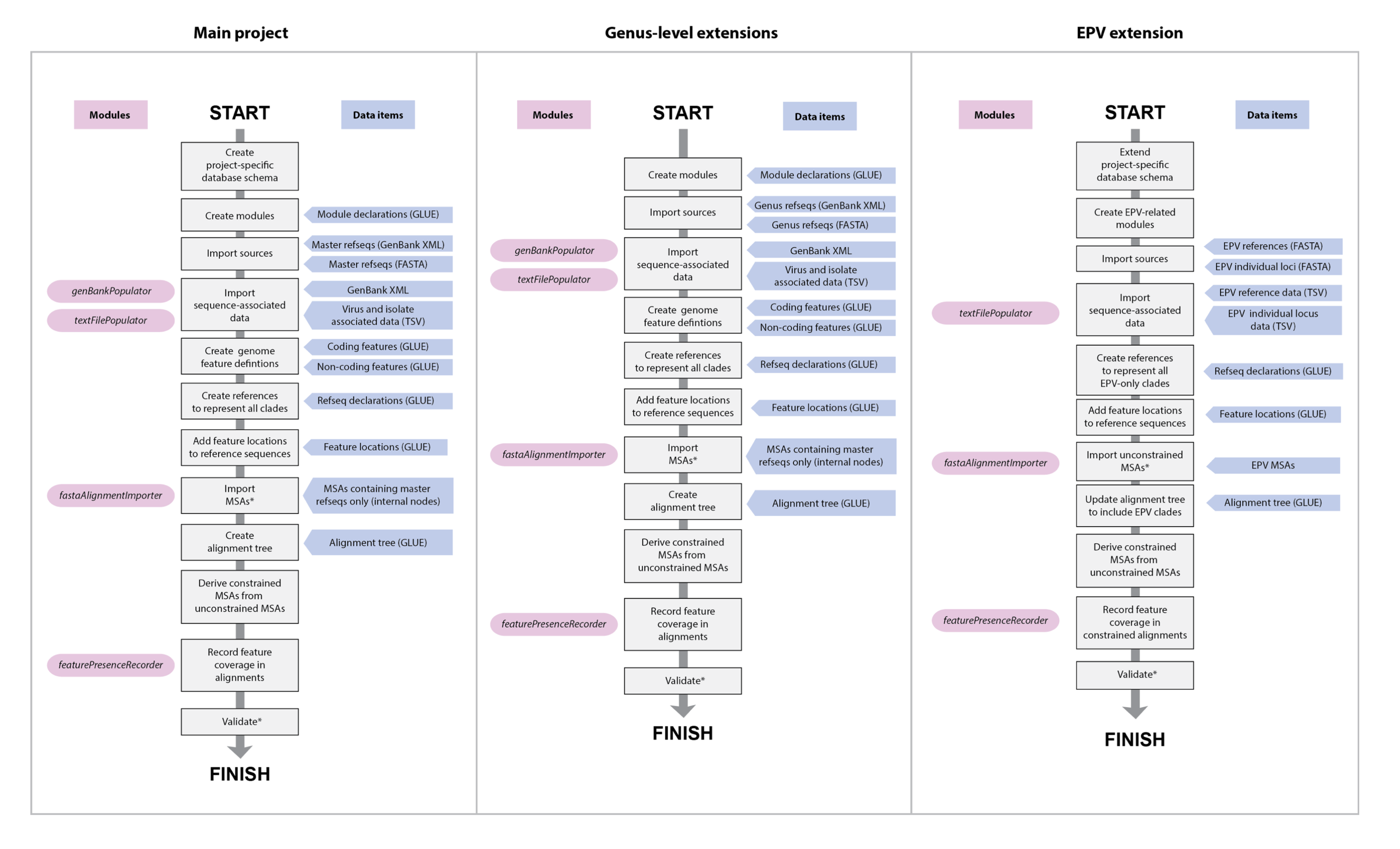


**Figure S2. The Parvovirus-GLUE resource build process.** Flowchart showing the process through which (i) the core project database is constructed in the Parvovirus-GLUE resource (left); (ii) the Parvovirus-GLUE project database is extended via incorporation of genus-level project layers; (iii) the Parvovirus-GLUE project database is extended through the addition of EPV-specific project layers for individual parvovirus genera. The data underlying this figure can be found in [https://zenodo.org/record/6968218](https://zenodo.org/record/6968218#.Yu115vHMIUY)
