# Supplementary material for: Comparative analysis reveals the long-term coevolutionary history of parvoviruses and vertebrates
Source: PLoS Biol. 2022 Nov 29;20(11):e3001867. doi: 10.1371/journal.pbio.3001867 (PMC9707805; doi:10.1371/journal.pbio.3001867)
Supplement: S3 Fig — (DOCX) [file pbio.3001867.s003.docx]

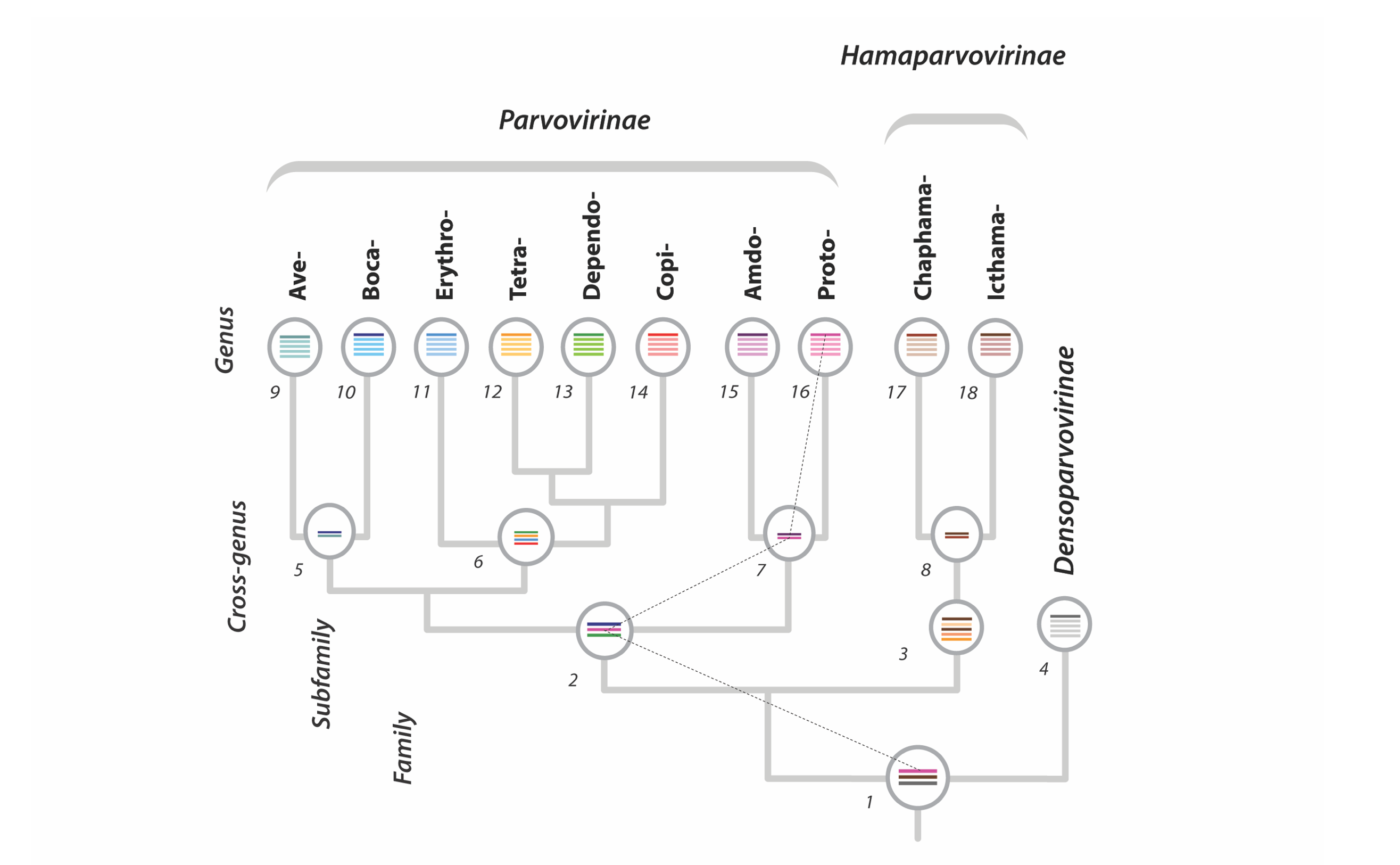


**Figure S3. A constrained alignment tree for the *Parvoviridae*.** The schematic shows a represention of the constrained alignment tree data structure implemented in the *Parvoviridae*-GLUE resource. It comprises a set of multiple sequence alignments (MSAs) that are hierarchically linked to reflect taxonomic relationships and represents the entire *Parvoviridae* family (monotypic genera are not shown). Numbers on nodes correspond to the MSA numbers shown in **Table 1**. The dashed line illustrates how the project master reference sequence, carnivore protoparvovirus 1 (CPV-1), is present in the family, subfamily, and cross-genus MSAs, as well in the genus-level MSA for the protoparvoviruses, and thus links MSAs from root to tip. Moreover, since the relationships of all *Parvovirinae* reference sequences to CPV are captured - either directly or indirectly - by this MSA set, all of the MSAs are linked. This approach effectively allows a single underlying MSA to be used to perform phylogenetic reconstructions across a range of taxonomic levels within the family. Furthermore, because each MSA is constrained to a master reference, a standardised genomic coordinate space is imposed on all parvovirus species genomes. The data underlying this figure can be found in [https://zenodo.org/record/6968218](https://zenodo.org/record/6968218#.Yu115vHMIUY)
