# Supplementary material for: Comparative analysis reveals the long-term coevolutionary history of parvoviruses and vertebrates
Source: PLoS Biol. 2022 Nov 29;20(11):e3001867. doi: 10.1371/journal.pbio.3001867 (PMC9707805; doi:10.1371/journal.pbio.3001867)
Supplement: S4 Fig — (DOCX) [file pbio.3001867.s004.docx]

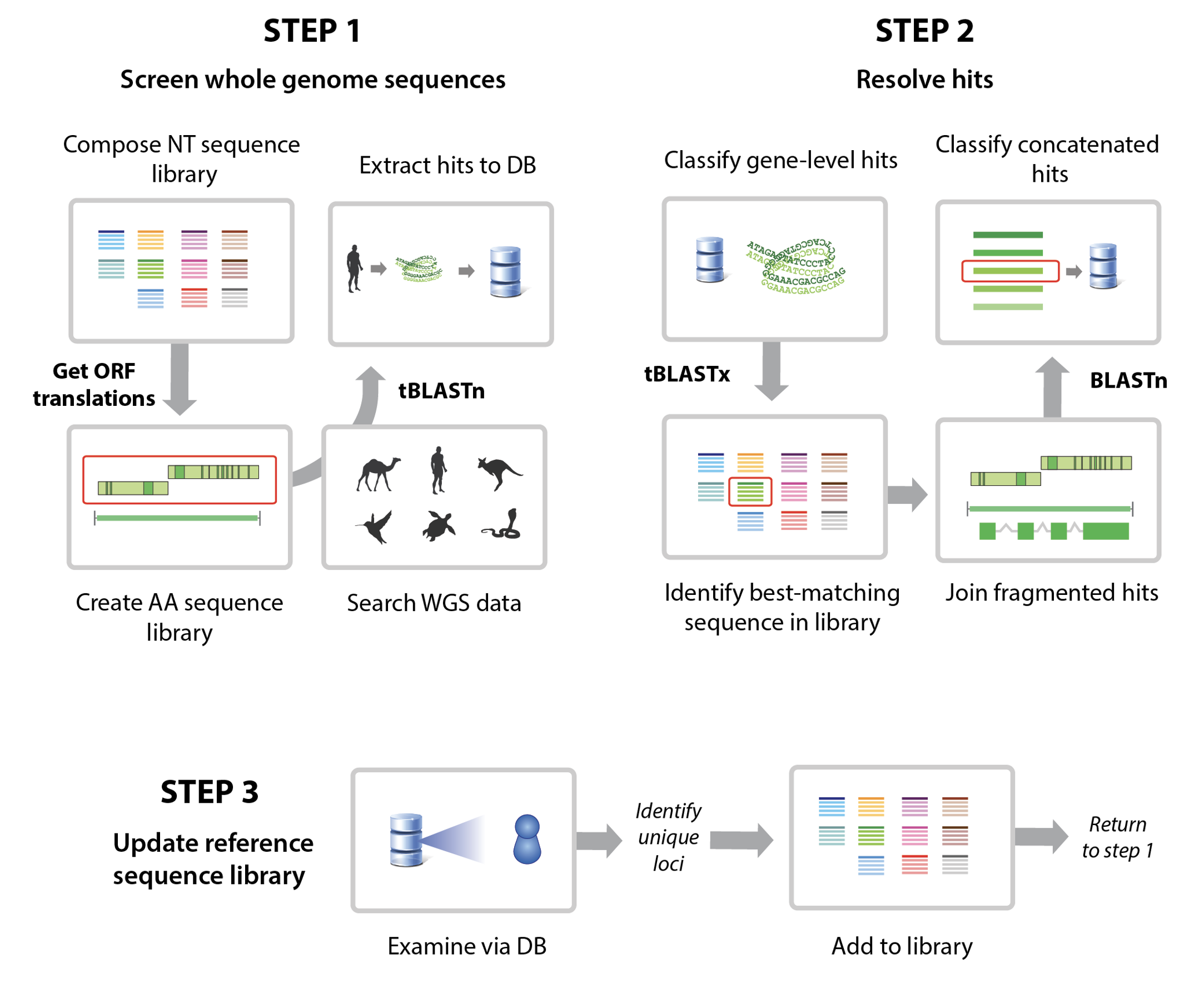


**Figure S4.** **Genome screening *in silico*.** The figure shows a schematic representation of the database-integrated genome-screening (DIGS) process used to identify EPV sequences.  **(1)** We collated a ‘reference sequence library’ representing all known parvovirus species and all known EPVs, and corresponding open reading frame (ORF) annotations. We used GLUE to derive a library of translated ORF sequences from these data and used these as ‘queries’ in tBLASTn-based searches of vertebrate WGS databanks. Hits were extracted to a relational database (DB). **(2)** Hits were initially classified by tBLASTx-based comparison to the translated ORF library, and these classifications were recorded, along with other information about the hit (species genome, and assembly version, hit sequence, coordinates and orientation) in a relational database. Hits that were within 1000bp of one another were concatenated and classified the via BLASTn-based comparison to a nucleotide-level reference library containing virus genomes and previously characterised EPVs. As we progressively identified novel EPV loci we incorporated them into this library. **(3)** Database-assisted analysis of similarity scores – combined with *ad hoc* phylogenetic anayses – were used to filter hits and identify sets of orthologous EPV insertions. The software processes shown this figure are implemented in the DIGS tool, available via <https://zenodo.org/record/6855611>
