# Supplementary material for: Comparative analysis reveals the long-term coevolutionary history of parvoviruses and vertebrates
Source: PLoS Biol. 2022 Nov 29;20(11):e3001867. doi: 10.1371/journal.pbio.3001867 (PMC9707805; doi:10.1371/journal.pbio.3001867)
Supplement: S5 Fig — Flowcharts showing the process through which maximum likelihood phylogenies of endogenous parvoviral elements (EPVs) were constructed using Parvovirus-GLUE. The data underlying this figure can be found in https://zenodo.org/record/6968218. (DOCX) [file pbio.3001867.s005.docx]

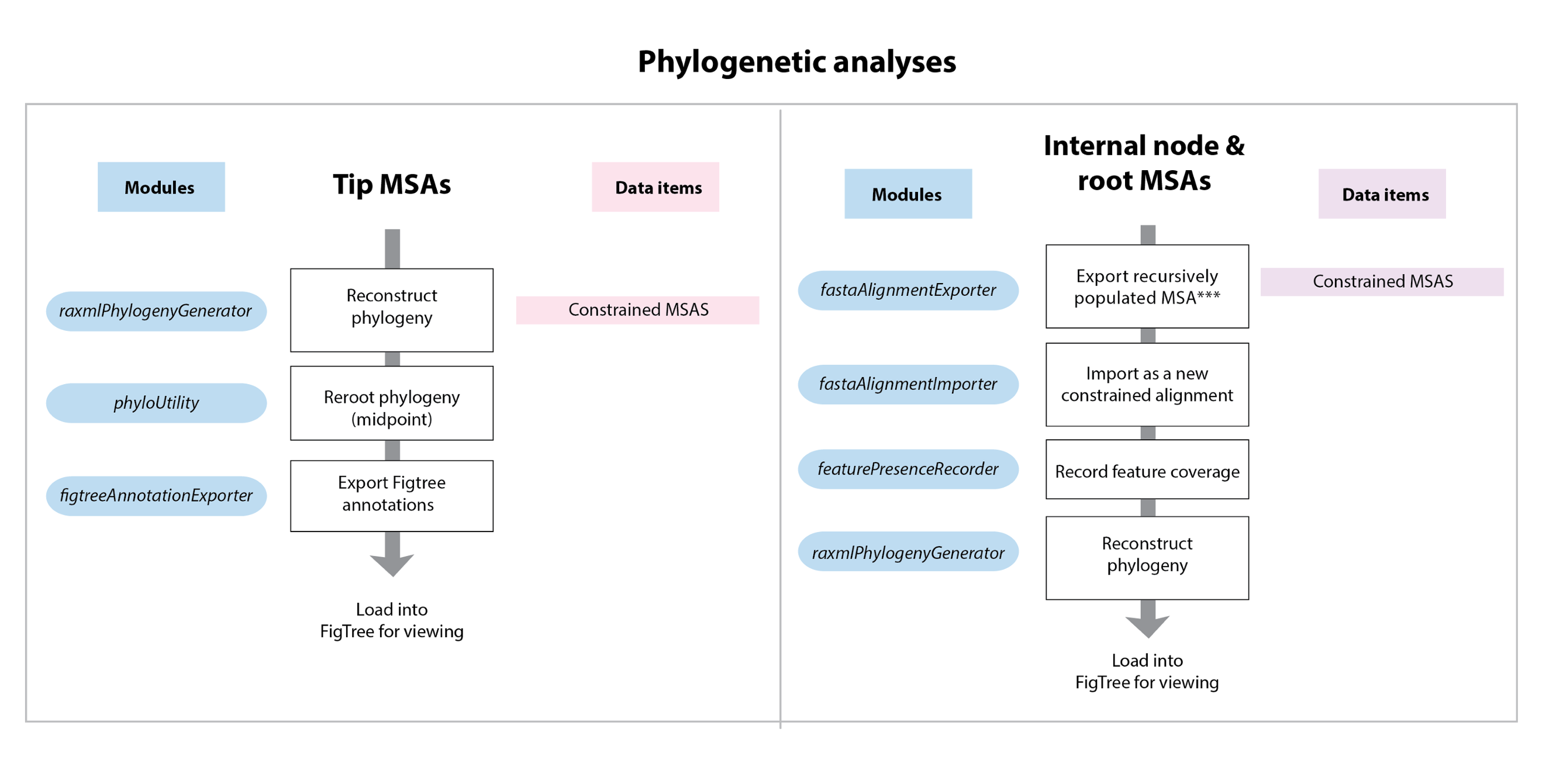


**Figure S5. Phylogeny construction using Parvovirus-GLUE.** Flowcharts showing the process through which maximum likelihood phylogenies of endogenous parvoviral elements (EPVs) were constructed using Parvovirus-GLUE. The data underlying this figure can be found in [https://zenodo.org/record/6968218](https://zenodo.org/record/6968218#.Yu115vHMIUY)
