# Supplementary material for: Comparative analysis reveals the long-term coevolutionary history of parvoviruses and vertebrates
Source: PLoS Biol. 2022 Nov 29;20(11):e3001867. doi: 10.1371/journal.pbio.3001867 (PMC9707805; doi:10.1371/journal.pbio.3001867)
Supplement: S6 Fig — (DOCX) [file pbio.3001867.s006.docx]

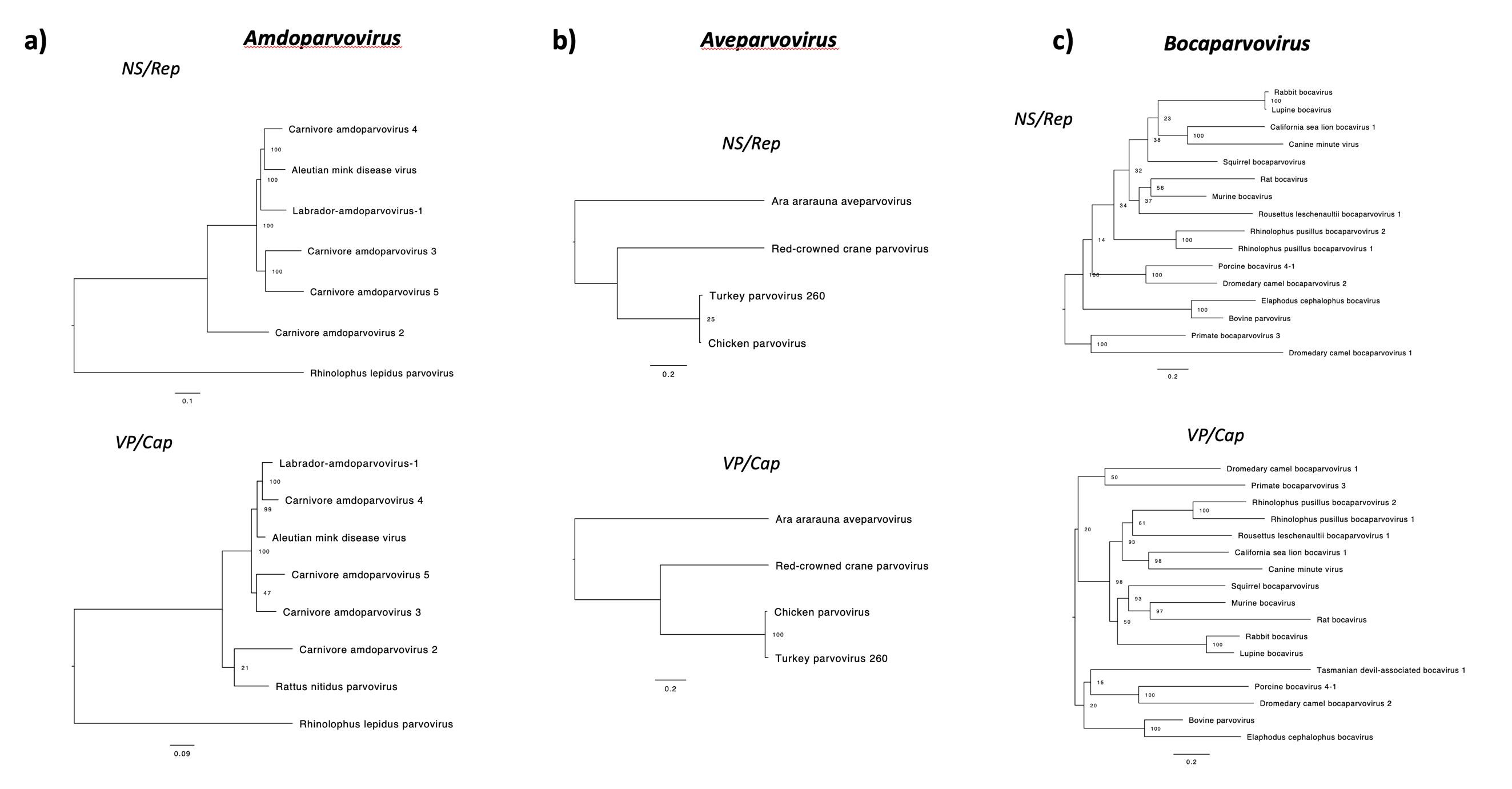


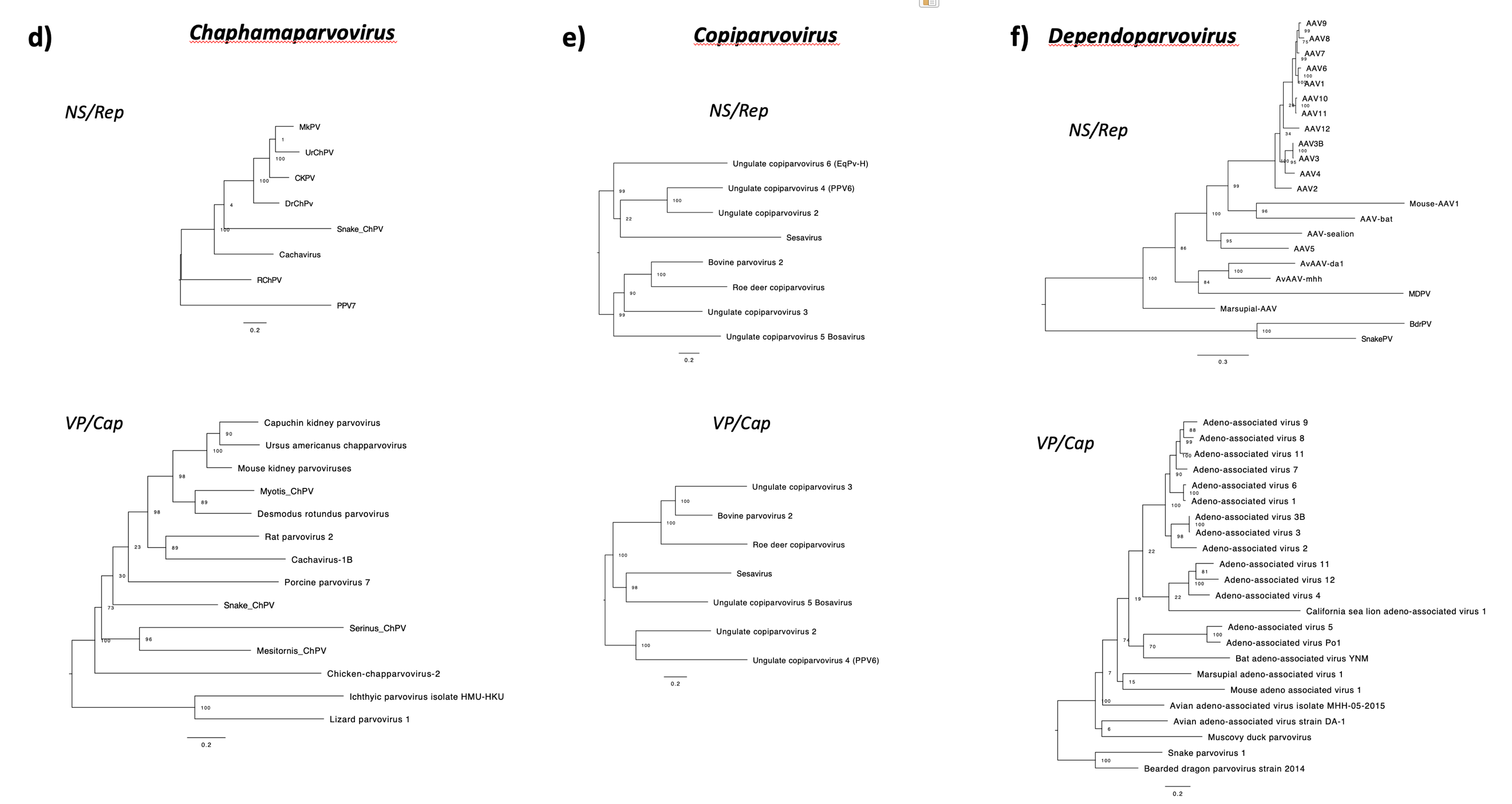


**Figure S6. Comprehensive phylogenetic analysis of subfamily *Parvovirinae* using virus sequences only.** Panels (a-g) show maximum likelihood phylogenies showing evolutionary relationships within parvovirus genera infecting vertebrates. Phylogenies were generated from codon-level multiple sequence alignments of NS/Rep and VP/Cap gene sequences, as follows: (a) *Amdoparvovirus*; (b) *Aveparvovirus*; (c) *Bocaparvovirus*; (d) *Chaphamaparvovirus*; (e) *Copiparvovirus*; (f) *Dependoparvovirus*; (g) *Erythroparvovirus*; (g) *Protoparvovirus*; (h) *Tetraparvovirus*. Trees for individual EPV loci are available in the Parvovirus-GLUE online resource [1]. The data underlying this figure can be found in [https://zenodo.org/record/6968218](https://zenodo.org/record/6968218#.Yu115vHMIUY)
