# Supplementary material for: Comparative analysis reveals the long-term coevolutionary history of parvoviruses and vertebrates
Source: PLoS Biol. 2022 Nov 29;20(11):e3001867. doi: 10.1371/journal.pbio.3001867 (PMC9707805; doi:10.1371/journal.pbio.3001867)
Supplement: S8 Fig — (DOCX) [file pbio.3001867.s008.docx]

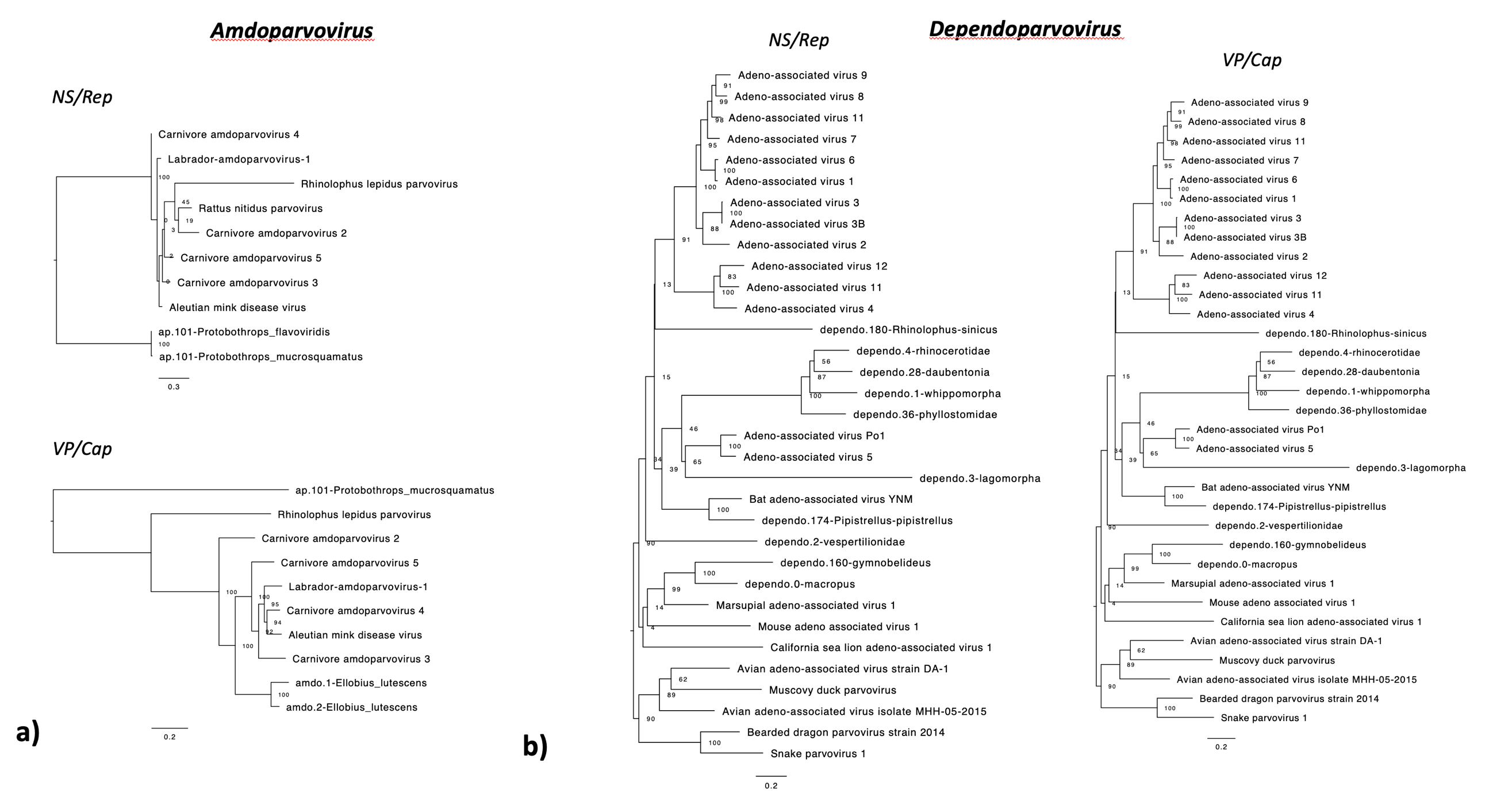


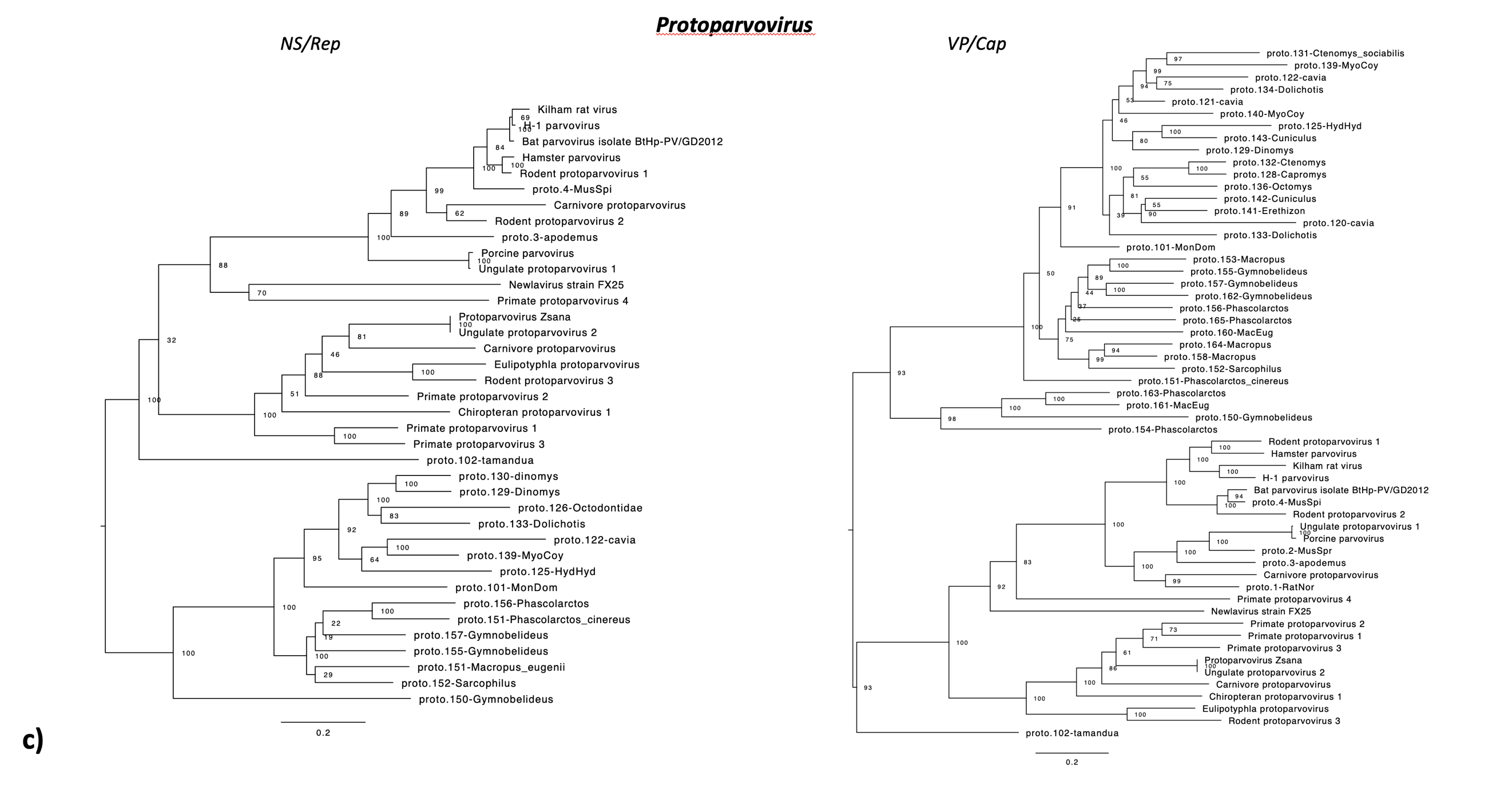


**Figure S8. Comprehensive phylogenetic analysis of subfamily *Parvovirinae* including virus and EPV sequences.** Panels (a-g) display maximum likelihood phylogenies showing evolutionary relationships within parvovirus genera infecting vertebrates. Phylogenies were generated from codon- level multiple sequence alignments of NS/Rep and VP/Cap gene sequences, as follows: (a) *Amdoparvovirus*; (b) *Dependoparvovirus*; (c) *Protoparvovirus*. The data underlying this figure can be found in [https://zenodo.org/record/6968218](https://zenodo.org/record/6968218#.Yu115vHMIUY)
