# Supplementary material for: Comparative analysis reveals the long-term coevolutionary history of parvoviruses and vertebrates
Source: PLoS Biol. 2022 Nov 29;20(11):e3001867. doi: 10.1371/journal.pbio.3001867 (PMC9707805; doi:10.1371/journal.pbio.3001867)
Supplement: S9 Fig — (DOCX) [file pbio.3001867.s009.docx]

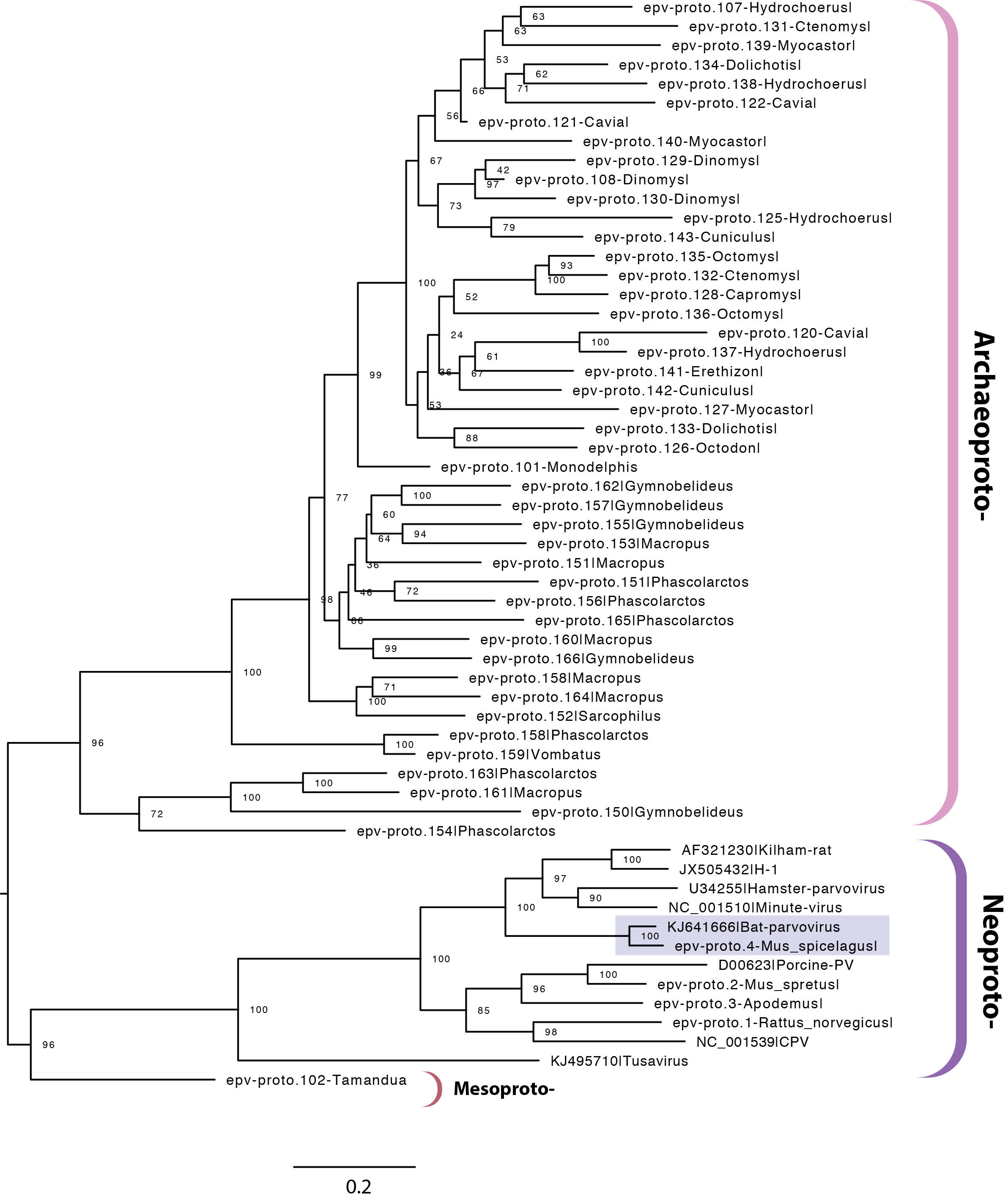


**Figure S9. Evolution of protoparvoviruses.** A maximum likelihood phylogenetic tree, based on an alignment of VP/capsid polypeptide sequences (544 amino acid residues) and showing the reconstructed evolutionary relationships between protoparvoviruses and protoparvoviruses-derived EPVs. The phylogeny was reconstructed using RAXML and the LG substitution model. Scale bar shows evolutionary distance in substitutions per site. Asterisks indicate nodes with >70% bootstrap support (1000 replicates). The close relationship between a ‘neoprotoparvovirus’-derived EPV identified in this study (EPV-Proto.4-MusSpre) and a recently identified, bat-associated protoparvovirus (“Pomona roundleaf bat protoparvovirus”), is highlighted (lilac rectangle)**.** The data underlying this figure can be found in [https://zenodo.org/record/6968218](https://zenodo.org/record/6968218#.Yu115vHMIUY)
