# Supplementary material for: Comparative analysis reveals the long-term coevolutionary history of parvoviruses and vertebrates
Source: PLoS Biol. 2022 Nov 29;20(11):e3001867. doi: 10.1371/journal.pbio.3001867 (PMC9707805; doi:10.1371/journal.pbio.3001867)
Supplement: S10 Fig — (DOCX) [file pbio.3001867.s010.docx]

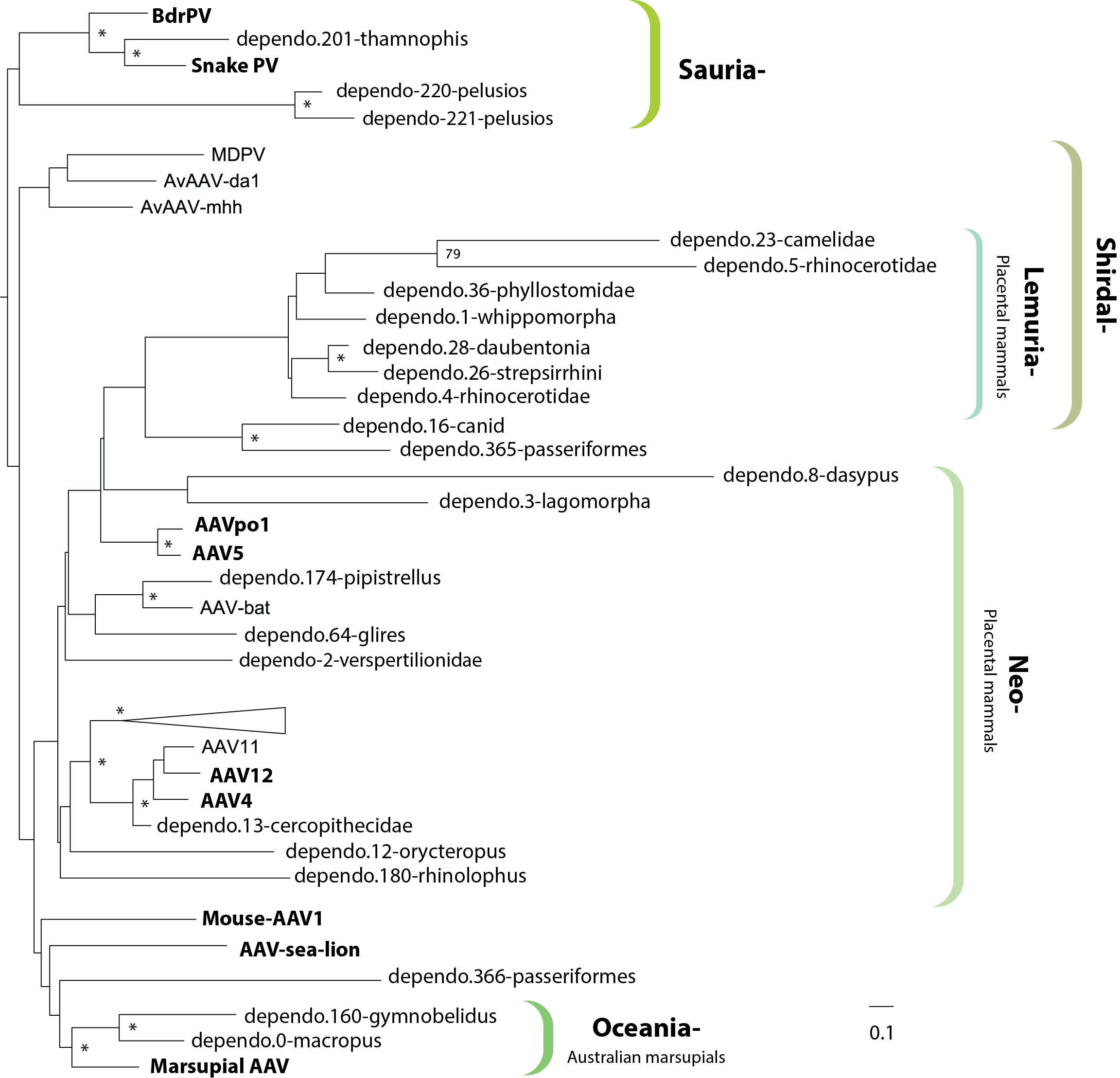


**Figure S10. Dependoparvovirus VP/capsid phylogeny.** A maximum likelihood phylogenetic tree, based on an alignment of VP/capsid polypeptide sequences (195 amino acid residues) and showing the reconstructed evolutionary relationships between dependoparvoviruses and dependoparvoviruses-derived EPVs. The phylogeny was reconstructed using RaXML and the LG model of amino acid substitution. Asterisks indicate nodes with support >70% (1000 bootstrap replicates). The data underlying this figure can be found in [https://zenodo.org/record/6968218](https://zenodo.org/record/6968218#.Yu115vHMIUY)
