# Supplementary material for: Comparative analysis reveals the long-term coevolutionary history of parvoviruses and vertebrates
Source: PLoS Biol. 2022 Nov 29;20(11):e3001867. doi: 10.1371/journal.pbio.3001867 (PMC9707805; doi:10.1371/journal.pbio.3001867)
Supplement: S11 Fig — (DOCX) [file pbio.3001867.s011.docx]

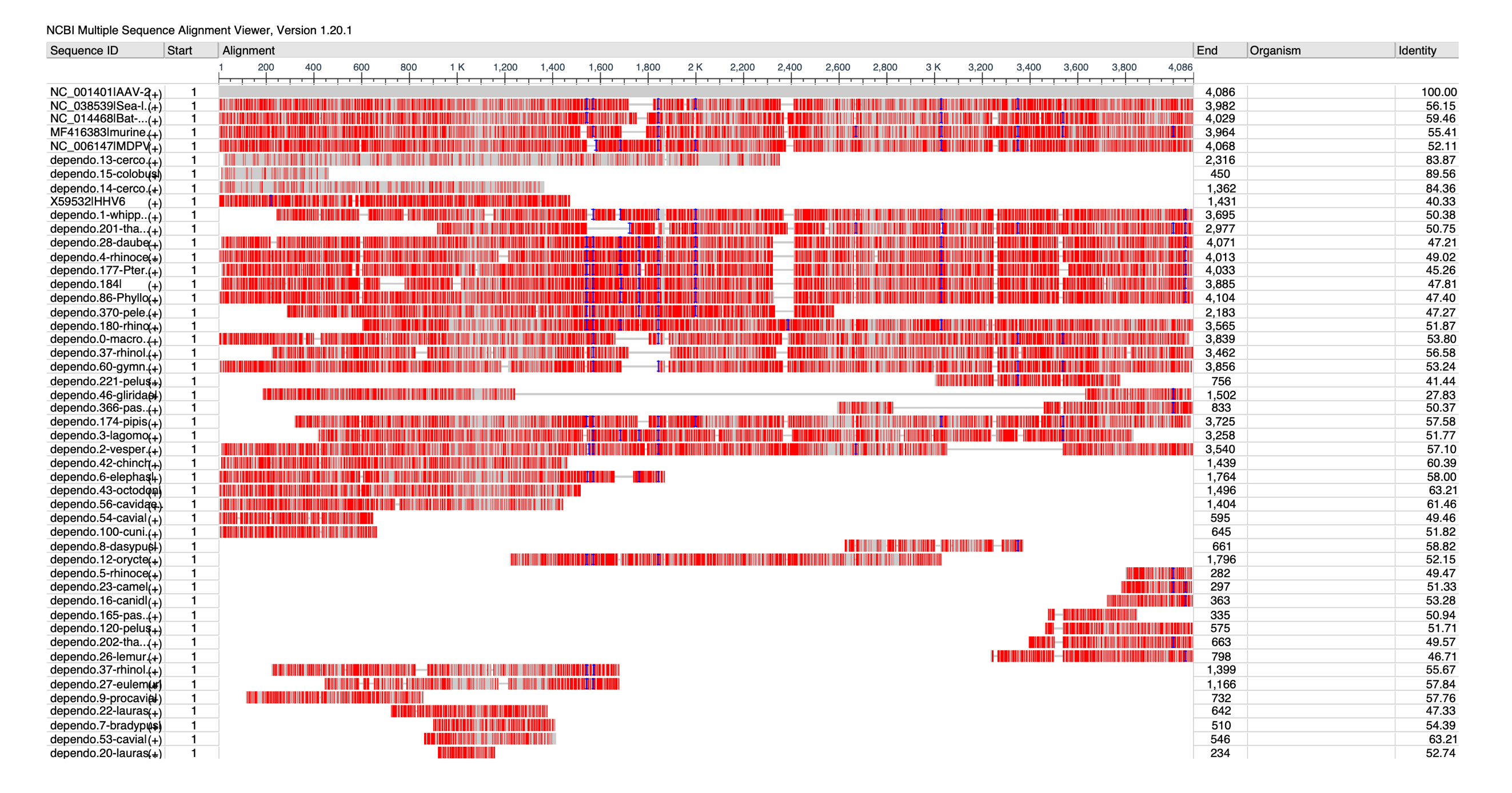


**Figure S11. Multiple sequence alignments of EPV and virus sequences.** Figure summarising dependoparvovirus EPV coverage relative to a master reference sequence (adeno-associated virus 2). The data underlying this figure can be found in [https://zenodo.org/record/6968218](https://zenodo.org/record/6968218#.Yu115vHMIUY)
