# Supplementary material for: Comparative analysis reveals the long-term coevolutionary history of parvoviruses and vertebrates
Source: PLoS Biol. 2022 Nov 29;20(11):e3001867. doi: 10.1371/journal.pbio.3001867 (PMC9707805; doi:10.1371/journal.pbio.3001867)
Supplement: S12 Fig — (DOCX) [file pbio.3001867.s012.docx]

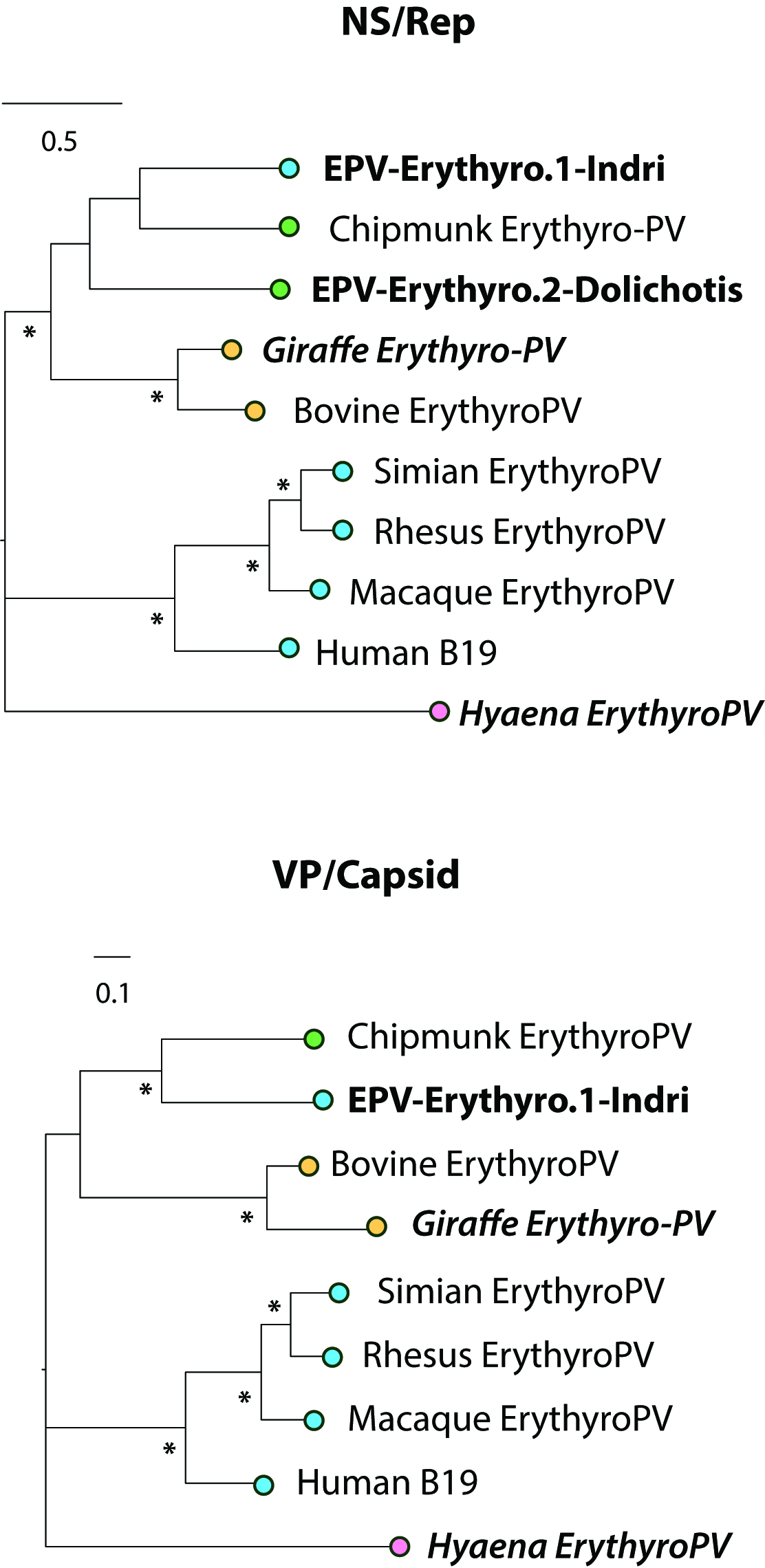


**Figure S12. Evolution of the *Erythroparvovirus* genus**

Maximum likelihood phylogenetic trees showing the reconstructed evolutionary relationships between erythroparvoviruses and erythroparvovirus-derived EPVs. The Rep/NS phylogeny (left) was constructed using a polypeptide-level multiple sequence alignment (MSA) spanning 500 amino acid residues (substitution model= LG likelihood). The VP/Capsid phylogeny (right) was constructed using a multiple sequence alignment (MSA) spanning 780 amino acid residues (substitution model= LG likelihood). Scale bars show evolutionary distance in substitutions per site. Asterisks indicate nodes with >70% bootstrap support (1000 replicates). Bold taxa labels indicate EPV taxa, newly characterised viral taxa are shown in bold italic text while previously characterised viral taxa are shown in regular text. Coloured circles at leaf nodes indicate the taxonomic order of mammals with which the EPV/virus species is associated, as follows: pink=Carnivora; blue=Primates; yellow=Artiodactyla; green=Rodents. The data underlying this figure can be found in [https://zenodo.org/record/6968218](https://zenodo.org/record/6968218#.Yu115vHMIUY)
