# Supplementary material for: Comparative analysis reveals the long-term coevolutionary history of parvoviruses and vertebrates
Source: PLoS Biol. 2022 Nov 29;20(11):e3001867. doi: 10.1371/journal.pbio.3001867 (PMC9707805; doi:10.1371/journal.pbio.3001867)
Supplement: S13 Fig — (DOCX) [file pbio.3001867.s013.docx]

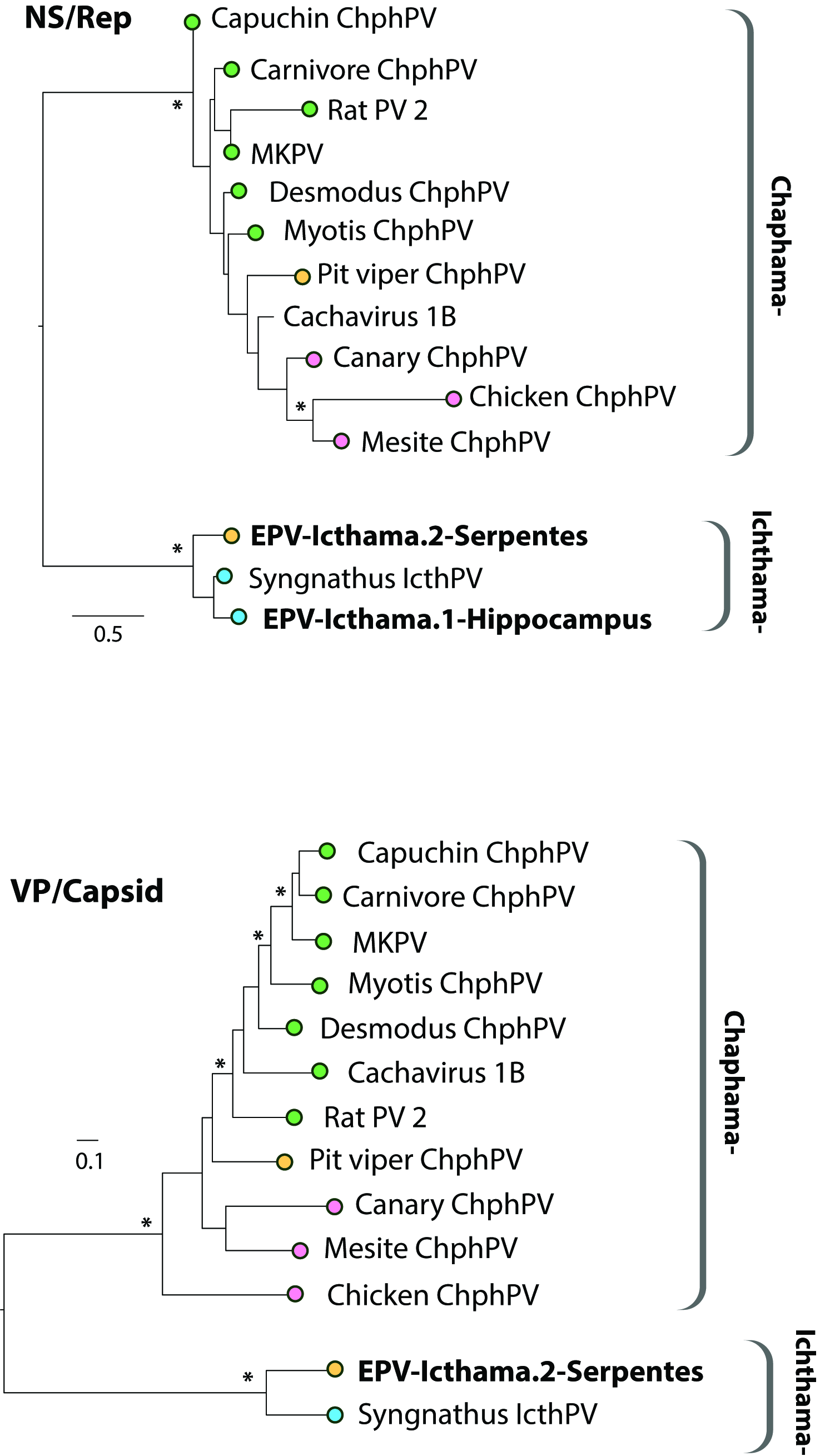


**Figure S13. Evolutionary relationships of vertebrate hamaparvoviruses.** Maximum likelihood phylogenetic trees showing the reconstructed evolutionary relationships between contemporary vertebrate hamaparvoviruses and hamaparvovirus-derived EPVs. The Rep/NS phylogeny (left) was constructed using a multiple sequence alignment (MSA) spanning 580 amino acid residues (substitution model= LG likelihood). The VP/Capsid phylogeny (right) was constructed using a multiple sequence alignment (MSA) spanning 450 amino acid residues (substitution model= LG likelihood). Scale bars show evolutionary distance in substitutions per site. Asterisks indicate nodes with >70% bootstrap support (1000 replicates). Coloured circles indicate host associations as follows: blue=fish; pink=birds; yellow=reptiles; green=mammals. Bold taxa labels indicate EPV taxa, while viral taxa are shown in regular text. The data underlying this figure can be found in [https://zenodo.org/record/6968218](https://zenodo.org/record/6968218#.Yu115vHMIUY)
