# Supplementary material for: Comparative analysis reveals the long-term coevolutionary history of parvoviruses and vertebrates
Source: PLoS Biol. 2022 Nov 29;20(11):e3001867. doi: 10.1371/journal.pbio.3001867 (PMC9707805; doi:10.1371/journal.pbio.3001867)
Supplement: S1 Table — (DOCX) [file pbio.3001867.s014.docx]

**Table S1. Parvovirus reference genomes included in Parvovirus-GLUE**

| **Accession^a^** | **Abbreviation ^b^** | **Full name ^c^** | **Isolation host ^d^** |
| --- | --- | --- | --- |
|  |  |  |  |
| ***Parvovirinae: Amdoparvovirus*** | | | |
| NC 001662* | AMDV | Aleutian mink disease virus | *Neovison vison* |
| NC 038533 | GFAV | Carnivore amdoparvovirus 1 | *Urocyon cinereoargenteus* |
| MT770848 | LAPV | Carnivore amdoparvovirus 1 | *Martes americana* |
| NC 025825 | RFAV | Carnivore amdoparvovirus 1 | *Nyctereutes procyonoides* |
| NC 031751 | RpAPV | Carnivore amdoparvovirus 1 | *Ailurus fulgens* |
| NC 034445 | SKAV | Carnivore amdoparvovirus 1 | *Mephitis mephitis* |
|  |  |  |  |
| ***Parvovirinae: Artiparvovirus*** | | | |
| NC 016752* | ArtiPV | Artibeus jamaicensis parvovirus 1 | *Artibeus jamaicensis* |
|  |  |  |  |
| ***Parvovirinae: Aveparvovirus*** | | | |
| NC 024452* | ChPV | Chicken parvovirus | *Gallus gallus* |
| NC 040672 | CraneAvePV | Red-crowned crane parvovirus | *Grus japonensis* |
| MW046460 | MacawAvePV | Ara ararauna aveparvovirus | *Ara ararauna* |
| NC 038534 | TurkeyAvePV | Turkey parvovirus 260 | *Meleagris gallopavo* |
|  |  |  |  |
| ***Parvovirinae: Bocaparvovirus*** | | | |
| NC 001540* | BPV | Bovine parvovirus | *Bos taurus* |
| NC 004442 | CanFam-CarBV1 | Canine minute virus | *Canis familiaris* |
| MF682925 | Rol-BtBoV1 | Rousettus leschenaultii bocaparvovirus 1 | *Rousettus leschenaultii* |
| MF682922 | Rp-BtBoV1 | Rhinolophus pusillus bocaparvovirus 1 | *Rhinolophus pusillus* |
| MF682923 | Rp-BtBoV2 | Rhinolophus pusillus bocaparvovirus 2 | *Rhinolophus pusillus* |
| JF429835 | Sus-ArtBoV4-1 | Porcine bocavirus 4-1 | *Sus scrofa* |
| JN420361 | Zac-PnBov1 | California sea lion bocavirus 1 | *Zalophus californianus* |
|  |  |  |  |
| ***Parvovirinae: Copiparvovirus*** | | | |
| NC 006259* | BPV2 | Bovine parvovirus 2 | *Bos taurus* |
| NC 031959 | Bosavirus | Ungulate copiparvovirus 5 | *Bos taurus* |
| MN615703 | BosCopiPV | Ungulate copiparvovirus 3 | *Bos taurus* |
| MW256660 | EqPv-H | Ungulate copiparvovirus 6 | *Equus caballus* |
| NC 014665 | PPV4 | Ungulate copiparvovirus 2 | *Sus scrofa* |
| NC 023860 | PPV6 | Ungulate copiparvovirus 4 | *Sus scrofa* |
| NC 055518 | Roe | Roe deer copiparvovirus | *Ixodes ricinus* |
| KM035804 | SesaPv | Sesavirus | *sea lion* |
|  |  |  |  |
| ***Parvovirinae: Dependoparvovirus*** | | | |
| NC 001401* | AAV2 | Adeno-associated virus 2 | *Homo sapiens* |
| NC 014468 | AAV-bat | Bat adeno-associated virus YNM | *Myotis ricketti* |
| NC 038539 | AAV-sealion | California sea lion AAV 1 | *Zalophus californianus* |
| NC 002077 | AAV1 | Adeno-associated virus 1 | *Homo sapiens* |
| LY408697 | AAV10 | Adeno-associated virus 11 | *Homo sapiens* |
| AY631966 | AAV11 | Adeno-associated virus 11 | *Homo sapiens* |
| DQ813647 | AAV12 | Adeno-associated virus 12 | *Homo sapiens* |
| NC 001729 | AAV3 | Adeno-associated virus 3 | *Homo sapiens* |
| AF028705 | AAV3B | Adeno-associated virus 3B | *Homo sapiens* |
| NC 001829 | AAV4 | Adeno-associated virus 4 | *Homo sapiens* |
| NC 006152 | AAV5 | Adeno-associated virus 5 | *Homo sapiens* |
| AF028704 | AAV6 | Adeno-associated virus 6 | *Homo sapiens* |
| NC 006260 | AAV7 | Adeno-associated virus 7 | *Macaca mulatta* |
| NC 006261 | AAV8 | Adeno-associated virus 8 | *Macaca mulatta* |
| AX753250 | AAV9 | Adeno-associated virus 9 | *Homo sapiens* |
| FJ688147 | AAVpo1 | Adeno-associated virus Po1 | *Sus domestica* |
| NC 004828 | AvAAV-atcc | Avian AAV ATCC VR-865 | *Gallus gallus* |
| NC 006263 | AvAAV-da1 | Avian AAV strain DA-1 | *Gallus gallus* |
| NC 040671 | AvAAV-mhh | Avian AAV isolate MHH-05-2015 | *Cairina moschata* |
| NC 005889 | BAAV | Bovine adeno-associated virus | *Bos taurus* |
| NC 027429 | BdrPV | Bearded dragon parvovirus | *Pogona vitticeps* |
| DQ335246 | CAV | Caprine adeno-associated virus | *Capra hircus* |
| NC 001701 | GPV | Goose parvovirus | *Anser anser* |
| MK026553 | Marsupial-AAV | Marsupial adeno-associated virus 1 | *marsupial* |
| NC 006147 | MDPV | Muscovy duck parvovirus | *Cairina moschata* |
| MF416383 | Mouse-AAV1 | Mouse adeno associated virus 1 | *Mus musculus* |
| MF416384 | Mouse-AAV2 | Mouse adeno associated virus 2 | *Mus musculus* |
| KT984498 | NHP-AAV6 | NHP Adeno-associated virus 6 | *Macaca mulatta* |
| NC 006148 | SnakePV | Snake parvovirus 1 | *Python regius* |
|  |  |  |  |
| ***Parvovirinae: Erythroparvovirus*** | | | |
| NC 000883* | B19 | Human parvovirus B19 | *Homo sapiens* |
| AF406967 | BPV3 | Ungulate erythroparvovirus 1 | *Bos taurus* |
| NC 038543 | ChpPV | Rodent erythroparvovirus 1 | *Tamias sibiricus* |
| NC 038542 | PmPV | Primate erythroparvovirus 4 | *Macaca nemestrina* |
| NC 038541 | RmPV | Primate erythroparvovirus 3 | *Macaca mulatta* |
| KF373759 | SePV | Pinniped erythroparvovirus 1 | *Phoca vitulina* |
| NC 038540 | SPV | Primate erythroparvovirus 2 | *Macaca fascicularis* |
|  |  |  |  |
| ***Parvovirinae: Loriparvovirus*** | | | |
| KP120516* | SlPV | Slow loris parvovirus 1 | *Nycticebus coucang* |
|  |  |  |  |
| ***Parvovirinae: Protoparvovirus*** | | | |
| NC 001539* | CPV | Carnivore protoparvovirus | *Canis familiaris* |
| NC 029797 | BtBuV1 | Chiropteran protoparvovirus 1 | *Pteropus vampyrus* |
| KJ641666 | BtHp-PV | Bat parvovirus isolate BtHp-PV/GD2012 | *Hipposideros pomona* |
| NC 038544 | BuV1a | Primate protoparvovirus 1 | *Unknown* |
| NC 039050 | CutaV | Primate protoparvovirus 3 | *Homo sapiens* |
| NC 026815 | EuPV | Eulipotyphla protoparvovirus | *Crocidura hirta* |
| JX505432 | H1 | H-1 parvovirus | *Rattus norvegicus* |
| U34255 | HV | Hamster parvovirus | *Mesocricetus auratus* |
| AF321230 | KilhamRat | Kilham rat virus | *Rattus norvegicus* |
| NC 001510 | MVM | Rodent protoparvovirus 1 | *Mus musculus* |
| NC 043446 | PBuV | Ungulate protoparvovirus 2 | *Sus scrofa* |
| D00623 | PPV | Porcine parvovirus | *Sus scrofa* |
| NC 001718 | PPV | Ungulate protoparvovirus 1 | *Sus scrofa* |
| NC 028650 | RatBuV | Rodent protoparvovirus 3 | *Unknown rat* |
| NC 038545 | RPV1 | Rodent protoparvovirus 2 | *Rattus norvegicus* |
| NC 030837 | SoPV | Carnivore protoparvovirus | *Enhydra lutris* |
| KJ495710 | TuV | Primate protoparvovirus 4 | *Unknown* |
| NC 039049 | WuBuV1 | Primate protoparvovirus 2 | *Macaca mulatta* |
| KT965075 | Zsana | Protoparvovirus Zsana | *Sus scrofa* |
| NC 007018* | PARV4 | Human parvovirus 4 | *Homo sapiens* |
| EU200669 | BovineHoko1 | Bovine hokovirus 1 | *Bos taurus* |
| HQ113143 | ChimpPV4 | Chimpanzee parvovirus 4 | *Pan troglodytes* |
| MG745671 | DdPV | Didelphimorphs tetraparvovirus | *Didelphis albiventris* |
| NC 031670 | DeerTetraPV | Deer tetraparvovirus | *Odocoileus hemionus* |
| NC 016744 | EhBtPV | Eidolon helvum parvovirus 1 | *Eidolon helvum* |
| JF504699 | OvineHoko1 | Ovine hokovirus 1 | *Ovis aries* |
| EU200677 | PorcineHoko1 | Porcine hokovirus | *Sus scrofa* |
| GU938300 | PorcineTetraPV | Porcine tetraparvovirus | *Sus scrofa* |
| MG745669 | RodentTetraPV | Rodent tetraparvovirus | *Bolomys lasiurus* |
|  |  |  |  |
| ***Hamaparvovirinae*** |  |  |  |
| MH670587* | MkPV | Mouse kidney parvoviruses | *Mus musculus* |
| MK448316 | Cachavirus | Cachavirus-1B | *Canis familiaris* |
| MG846443 | CChPV | Chicken-chapparvovirus-2 | *Gallus gallus* |
| MN265364 | CKPV | Capuchin kidney parvovirus | *Cebus capucinus* |
| NC 032097 | DrChPv | Desmodus rotundus parvovirus | *Desmodus rotundus* |
| NC 040562 | PPV7 | Porcine parvovirus 7 | *Sus scrofa* |
| KX272741 | RChPV | Rat parvovirus 2 | *Rattus norvegicus* |
| MN166196 | UrChPV | Ursus americanus chapparvovirus | *Ursus americanus* |
| MN049932* | SyngChPV | Syngnathus scovelli chapparvovirus | *Syngnathus scovelli* |
| MN162688 | IcthPV | Ichthyic parvovirus isolate HMU-HKU | *Tilapia* |
| NC 002190* | IHHN | Infectious hypodermal and hematopoietic necrosis virus |  |
|  |  |  |  |
| ***Densoparvovirinae*** |  |  |  |
| NC 004285* | AalDNV-2 | Aedes albopictus densovirus 2 | *Aedes albopictus* |
| NC 014357* | FCDNV | Fenneropenaeus chinensis hepatopancreatic densovirus | *Fenneropenaeus chinensis* |
| NC 038532* | AaDV | Asteroid aquambidensovirus 1 | *Asteroidea* |
| NC 005041* | BgDV | Blattella germanica densovirus 1 | *Blattella germanica* |
| NC 004287* | BmDNV-5 | Bombyx mori densovirus 5 | *Bombyx mori* |
| NC 022564* | AdMADV | Acheta domesticus mini ambidensovirus | *Acheta domesticus* |
| NC 000936* | PfDNV | Periplaneta fuliginosa densovirus | *Periplaneta fuliginosa* |
| NC 004286* | GmDV | Galleria mellonella densovirus | *Galleria mellonella* |
| NC 004289* | PcDV | Planococcus citri densovirus | *Planococcus citri* |
| NC 004284* | JcDNV | Junonia coenia densovirus | *Junonia coenia* |
|  |  |  |  |

**Footnote**: ^a^ NCBI GenBank accession number. ^b^ Abbreviated virus name. ^c^ Full virus name. ^d^ Latin binomial species name of animal virus was isolated from. * Asterisks indicate genomes chosen as ‘master references’ for taxonomic groups within family *Parvoviridae*.
