# Supplementary material for: Comparative analysis reveals the long-term coevolutionary history of parvoviruses and vertebrates
Source: PLoS Biol. 2022 Nov 29;20(11):e3001867. doi: 10.1371/journal.pbio.3001867 (PMC9707805; doi:10.1371/journal.pbio.3001867)
Supplement: S2 Table — (DOCX) [file pbio.3001867.s015.docx]

**Table S2. Parvovirus genome features defined in Parvovirus-GLUE**

| **Feature name** | **Parent feature name** | **Feature description** |
| --- | --- | --- |
|  |  |  |
| whole genome | - | Whole genome |
| X | whole genome | X protein |
| VP3 | whole genome | Capsid protein VP3 |
| VP2 | whole genome | Capsid protein VP2 |
| VP1 | whole genome | Capsid protein VP1 |
| Rep78 | whole genome | Rep 78 protein |
| Rep68 | whole genome | Rep 68 protein |
| Rep52 | whole genome | Rep 52 protein |
| Rep40 | whole genome | Rep 40 protein |
| ORF7 | whole genome | ORF7 protein |
| ORF6 | whole genome | ORF6 protein |
| ORF5 | whole genome | ORF5 protein |
| ORF4 | whole genome | ORF4 protein |
| ORF3 | whole genome | ORF3 protein |
| ORF2 | whole genome | ORF2 protein |
| ORF1 | whole genome | ORF1 protein |
| NS2 | whole genome | Replicase protein NS2 |
| NS1 | whole genome | Replicase protein NS1 |
| NS | whole genome | Replicase protein |
| NP1 | whole genome | NP1 protein |
| NP | whole genome | NP protein |
| M | whole genome | Mid-ORF |
| CP | whole genome | Capsid protein (Denso) |
| AAP | whole genome | Assembly activating protein |
| 5UTR | whole genome | 5 prime UTR |
| 3UTR | whole genome | 3 prime UTR |
| Beta-IX | VP1 | Beta sheet IX |
| Beta-I | VP1 | Beta sheet I |
| Beta-H | VP1 | Beta sheet H |
| Beta-G | VP1 | Beta sheet G |
| Beta-F | VP1 | Beta sheet F |
| Beta-E | VP1 | Beta sheet E |
| Beta-D | VP1 | Beta sheet D |
| Beta-C | VP1 | Beta sheet C |
| Beta-B | VP1 | Beta sheet B |
| ATPase-C | Rep78 | ATPase-C domain |
| ATPase-B | Rep78 | ATPase-B domain |
| ATPase-A | Rep78 | ATPase-A domain |
| 5IR | 5UTR | 5 prime inverted terminal repeat |
| 3IR | 3UTR | 3 prime inverted terminal repeat |
| VL VIII | VP1 | Variable loop VIII |
| VL VII | VP1 | Variable loop VII |
| VL VI | VP1 | Variable loop VI |
| VL V | VP1 | Variable loop V |
| VL IV | VP1 | Variable loop IV |
| VL III | VP1 | Variable loop III |
| VL II | VP1 | Variable loop II |
| VL I | VP1 | Variable loop I |
| VL alpha-1 | VP1 | Variable loop alpha 1 |
| RCR | Rep78 | Rolling-circle replication domain |
| PLA2 | VP1 | Phospholipase A2 domain |
|  |  |  |

**Footnote:** Abbreviations: NS=replicase protein; VP=capsid protein; ORF = open reading frame; UTR=untranslated region; IR=inverted repeated; AAP=assembly activating protein; NP=nucleoprotein; VL=variable loop.
