# Supplementary material for: Comparative analysis reveals the long-term coevolutionary history of parvoviruses and vertebrates
Source: PLoS Biol. 2022 Nov 29;20(11):e3001867. doi: 10.1371/journal.pbio.3001867 (PMC9707805; doi:10.1371/journal.pbio.3001867)
Supplement: S3 Table — (DOCX) [file pbio.3001867.s016.docx]

**Table S3. Locations of genome features within parvovirus reference sequences**

| **Reference name^a^** | **Accession^b^** | **Feature name** | **Start ^c^** | **End ^d^** | **Splice ^e^** |
| --- | --- | --- | --- | --- | --- |
|  |  |  |  |  |  |
| **Dependoparvovirus** |  |  |  |  |  |
| AAV2 | NC_001401 | Genome | 1 | 4679 | 1 |
| AAV2 | ‘’ | VP3 | 2809 | 4410 | 1 |
| AAV2 | ‘’ | VP2 | 2614 | 4410 | 1 |
| AAV2 | ‘’ | VP1 | 2203 | 4410 | 1 |
| AAV2 | ‘’ | Rep78 | 321 | 2186 | 1 |
| AAV2 | ‘’ | Rep68 | 321 | 1906 | 1 |
| AAV2 | ‘’ | Rep68 | 2228 | 2252 | 2 |
| AAV2 | ‘’ | Rep52 | 993 | 2186 | 1 |
| AAV2 | ‘’ | Rep40 | 993 | 2252 | 1 |
| AAV2 | ‘’ | Dependo_X | 3929 | 4396 | 1 |
| AAV2 | ‘’ | AAP | 2729 | 3343 | 1 |
| AAV2 | ‘’ | 5UTR | 1 | 320 | 1 |
| AAV2 | ‘’ | 3UTR | 4411 | 4679 | 1 |
| AAV2 | ‘’ | VL_III | 3343 | 3363 | 1 |
| AAV2 | ‘’ | VL_II | 3178 | 3192 | 1 |
| AAV2 | ‘’ | VL_I | 2986 | 3006 | 1 |
| AAV2 | ‘’ | RCR | 585 | 608 | 1 |
| AAV2 | ‘’ | PLA2 | 2332 | 2514 | 1 |
| AAV2 | ‘’ | Beta-F | 3403 | 3414 | 1 |
| AAV2 | ‘’ | Beta-E | 3214 | 3240 | 1 |
| AAV2 | ‘’ | Beta-D | 3112 | 3171 | 1 |
| AAV2 | ‘’ | Beta-C | 3028 | 3054 | 1 |
| AAV2 | ‘’ | Beta-B | 2914 | 2955 | 1 |
| AAV2 | ‘’ | ATPase-C | 1440 | 1586 | 1 |
| AAV2 | ‘’ | ATPase-B | 1440 | 1469 | 1 |
| AAV2 | ‘’ | ATPase-A | 1308 | 1370 | 1 |
| AAV-bat | NC_014468 | Genome | 1 | 4286 | 1 |
| AAV-bat | ‘’ | VP1 | 2059 | 4233 | 1 |
| AAV-bat | ‘’ | Rep78 | 200 | 2041 | 1 |
| AAV5 | NC_006152 | Genome | 1 | 4642 | 1 |
| AAV5 | ‘’ | VP1 | 2207 | 4381 | 1 |
| AAV5 | ‘’ | Rep78 | 359 | 2191 | 1 |
| AAV6 | AF028704 | Genome | 1 | 4683 | 1 |
| AAV6 | ‘’ | VP1 | 2208 | 4418 | 1 |
| AAV6 | ‘’ | Rep78 | 320 | 2191 | 1 |
| AAV7 | NC_006260 | Genome | 1 | 4721 | 1 |
| AAV7 | ‘’ | VP1 | 2222 | 4435 | 1 |
| AAV7 | ‘’ | Rep78 | 334 | 2205 | 1 |
| AAV8 | NC_006261 | Genome | 1 | 4393 | 1 |
| AAV8 | ‘’ | VP1 | 2121 | 4337 | 1 |
| AAV8 | ‘’ | Rep78 | 227 | 2104 | 1 |
| AAV9 | AX753250 | Genome | 1 | 4385 | 1 |
| AAV9 | ‘’ | VP1 | 2116 | 4329 | 1 |
| AAV9 | ‘’ | Rep78 | 228 | 2099 | 1 |
| CAV | DQ335246 | Genome | 1 | 4636 | 1 |
| CAV | ‘’ | VP1 | 2195 | 4375 | 1 |
| CAV | ‘’ | Rep78 | 347 | 2179 | 1 |
| GPV | NC_001701 | Genome | 1 | 5106 | 1 |
| GPV | ‘’ | VP1 | 2439 | 4637 | 1 |
| GPV | ‘’ | Rep78 | 537 | 2420 | 1 |
| GPV | ‘’ | 5UTR | 1 | 536 | 1 |
| GPV | ‘’ | 3UTR | 4638 | 5106 | 1 |
| GPV | ‘’ | 5IR | 1 | 457 | 1 |
| GPV | ‘’ | 3IR | 4678 | 5106 | 1 |
| MAAV | MK026553 | Genome | 1 | 3472 | 1 |
| MAAV | ‘’ | VP1 | 1298 | 3472 | 1 |
| MAAV | ‘’ | Rep78 | 1 | 1287 | 1 |
| MDPV | NC_006147 | Genome | 1 | 5132 | 1 |
| MDPV | ‘’ | VP1 | 2450 | 4648 | 1 |
| MDPV | ‘’ | Rep78 | 548 | 2431 | 1 |
| MDPV | ‘’ | 5UTR | 1 | 547 | 1 |
| MDPV | ‘’ | 3UTR | 4649 | 5132 | 1 |
| MDPV | ‘’ | 5IR | 1 | 444 | 1 |
| MDPV | ‘’ | 3IR | 4663 | 5132 | 1 |
| Mouse-AAV1 | MF416383 | Genome | 1 | 4339 | 1 |
| Mouse-AAV1 | ‘’ | VP1 | 2049 | 4280 | 1 |
| Mouse-AAV1 | ‘’ | Rep78 | 314 | 2032 | 1 |
| SnakePV | NC_006148 | Genome | 1 | 4432 | 1 |
| SnakePV | ‘’ | VP1 | 2030 | 4210 | 1 |
| SnakePV | ‘’ | Rep78 | 324 | 2012 | 1 |
|  |  |  |  |  |  |
| **Amdoparvovirus** |  |  |  |  |  |
| AMDV | NC_001662 | Genome | 1 | 4801 | 1 |
| AMDV | ‘’ | VP1 | 2406 | 4349 | 1 |
| AMDV | ‘’ | Rep78 | 206 | 1978 | 1 |
| AMDV | ‘’ | M | 1993 | 2211 | 1 |
| BtRl-PV | KJ641663 | Genome | 1 | 4063 | 1 |
| BtRl-PV | ‘’ | VP1 | 1923 | 1932 | 1 |
| BtRl-PV | ‘’ | VP1 | 2007 | 4057 | 2 |
| BtRl-PV | ‘’ | Rep78 | 1 | 1716 | 1 |
| GFAV | NC_038533 | Genome | 1 | 4441 | 1 |
| GFAV | ‘’ | VP1 | 2148 | 2157 | 1 |
| GFAV | ‘’ | VP1 | 2236 | 4250 | 2 |
| GFAV | ‘’ | Rep78 | 110 | 1844 | 1 |
| GFAV | ‘’ | Rep78 | 1983 | 2155 | 2 |
| RFAV | NC_025825 | Genome | 1 | 4928 | 1 |
| RFAV | ‘’ | VP1 | 2244 | 2253 | 1 |
| RFAV | ‘’ | VP1 | 2326 | 4355 | 2 |
| RFAV | ‘’ | Rep78 | 237 | 1992 | 1 |
| RFAV | ‘’ | Rep78 | 2082 | 2251 | 2 |
| RpAPV | NC_031751 | Genome | 1 | 4662 | 1 |
| RpAPV | ‘’ | VP1 | 2309 | 4252 | 1 |
| RpAPV | ‘’ | Rep78 | 107 | 1894 | 1 |
| RtRn-ParV | KY432922 | Genome | 1 | 1331 | 1 |
| RtRn-ParV | ‘’ | VP1 | 288 | 1331 | 1 |
| SKAV | NC_034445 | Genome | 1 | 4242 | 1 |
| SKAV | ‘’ | VP1 | 2069 | 2078 | 1 |
| SKAV | ‘’ | VP1 | 2152 | 4175 | 2 |
| SKAV | ‘’ | Rep78 | 69 | 1824 | 1 |
| SKAV | ‘’ | Rep78 | 1907 | 2076 | 2 |
|  |  |  |  |  |  |
| **Bocaparvovirus** |  |  |  |  |  |
| BPV | NC_001540 | Genome | 1 | 5517 | 1 |
| BPV | ‘’ | VP1 | 3286 | 5307 | 1 |
| BPV | ‘’ | Rep78 | 740 | 2920 | 1 |
| BPV | ‘’ | NP | 2661 | 3302 | 1 |
| BPV | ‘’ | 5UTR | 1 | 739 | 1 |
| BPV | ‘’ | 3UTR | 5308 | 5517 | 1 |
| RolBtBoV1 | MF682925 | Genome | 1 | 5038 | 1 |
| RolBtBoV1 | ‘’ | VP1 | 2958 | 5030 | 1 |
| RolBtBoV1 | ‘’ | Rep78 | 208 | 2589 | 1 |
| RolBtBoV1 | ‘’ | NP | 2354 | 2971 | 1 |
| SusArtBoV41 | JF429835 | Genome | 1 | 5177 | 1 |
| SusArtBoV41 | ‘’ | VP1 | 3030 | 5084 | 1 |
| SusArtBoV41 | ‘’ | Rep78 | 232 | 2235 | 1 |
| SusArtBoV41 | ‘’ | NP | 2360 | 3040 | 1 |
| ZacPnBov1 | JN420361 | Genome | 1 | 5263 | 1 |
| ZacPnBov1 | ‘’ | VP1 | 3008 | 5167 | 1 |
| ZacPnBov1 | ‘’ | Rep78 | 258 | 2639 | 1 |
| ZacPnBov1 | ‘’ | NP | 2443 | 3024 | 1 |
|  |  |  |  |  |  |
| **Copiparvovirus** |  |  |  |  |  |
| BPV2 | NC_006259 | Genome | 1 | 5610 | 1 |
| BPV2 | ‘’ | VP1 | 2268 | 5384 | 1 |
| BPV2 | ‘’ | Rep78 | 306 | 1919 | 1 |
| BPV2 | ‘’ | 5UTR | 1 | 305 | 1 |
| BPV2 | ‘’ | 3UTR | 5383 | 5610 | 1 |
| Bosa | NC_031959 | Genome | 1 | 5371 | 1 |
| Bosa | ‘’ | VP1 | 2135 | 5242 | 1 |
| Bosa | ‘’ | Rep78 | 145 | 1911 | 1 |
| EqPvH | MW256660 | Genome | 1 | 4725 | 1 |
| EqPvH | ‘’ | VP1 | 1801 | 4725 | 1 |
| EqPvH | ‘’ | Rep78 | 1 | 1782 | 1 |
| PPV4 | NC_014665 | Genome | 1 | 5905 | 1 |
| PPV4 | ‘’ | VP1 | 3673 | 5859 | 1 |
| PPV4 | ‘’ | Rep78 | 849 | 2645 | 1 |
| PPV4 | ‘’ | ORF3 | 2795 | 3409 | 1 |
| PPV6 | NC_023860 | Genome | 1 | 6148 | 1 |
| PPV6 | ‘’ | VP1 | 2215 | 5784 | 1 |
| PPV6 | ‘’ | Rep78 | 219 | 2207 | 1 |
| Roe deer CoPV | NC_055518 | Genome | 1 | 6296 | 1 |
| Roe deer CoPV | ‘’ | VP1 | 2828 | 6109 | 1 |
| Roe deer CoPV | ‘’ | Rep78 | 670 | 2430 | 1 |
| SesaPV | KM035804 | Genome | 1 | 5049 | 1 |
| SesaPV | ‘’ | VP1 | 2060 | 4954 | 1 |
| SesaPV | ‘’ | Rep78 | 314 | 1975 | 1 |
| BosCopiPV | MN615703 | Genome | 1 | 5601 | 1 |
| BosCopiPV | ‘’ | VP1 | 2330 | 5599 | 1 |
| BosCopiPV | ‘’ | Rep78 | 365 | 2008 | 1 |
|  |  |  |  |  |  |
| **Aveparvovirus** |  |  |  |  |  |
| ChPV | NC_024452 | Genome | 1 | 5257 | 1 |
| ChPV | ‘’ | VP1 | 2998 | 5025 | 1 |
| ChPV | ‘’ | Rep78 | 411 | 2495 | 1 |
| ChPV | ‘’ | Ave_NP | 2696 | 3001 | 1 |
| ChPV | ‘’ | 5UTR | 1 | 145 | 1 |
| ChPV | ‘’ | 3UTR | 5026 | 5257 | 1 |
| TurkeyAvePV | NC_038534 | Genome | 1 | 4615 | 1 |
| TurkeyAvePV | ‘’ | VP1 | 2588 | 4615 | 1 |
| TurkeyAvePV | ‘’ | Rep78 | 1 | 2085 | 1 |
| TurkeyAvePV | ‘’ | Ave_NP | 2286 | 2591 | 1 |
| CraneAvePV | NC_040672 | Genome | 1 | 5456 | 1 |
| CraneAvePV | ‘’ | VP1 | 3386 | 5404 | 1 |
| CraneAvePV | ‘’ | Rep78 | 335 | 2377 | 1 |
| CraneAvePV | ‘’ | Ave_NP | 2950 | 3435 | 1 |
| MacawAvePV | MW046460 | Genome | 220 | 5458 | 1 |
| MacawAvePV | ‘’ | VP1 | 3307 | 5457 | 1 |
| MacawAvePV | ‘’ | Rep78 | 220 | 2118 | 1 |
| MacawAvePV | ‘’ | Ave_NP | 2151 | 3341 | 1 |
|  |  |  |  |  |  |
| **Erythroparvovirus** |  |  |  |  |  |
| B19 | NC_000883 | Genome | 1 | 5596 | 1 |
| B19 | ‘’ | VP1 | 2624 | 4969 | 1 |
| B19 | ‘’ | Rep78 | 616 | 2631 | 1 |
| B19 | ‘’ | X | 2874 | 3119 | 1 |
| B19 | ‘’ | ORF1 | 2084 | 2308 | 1 |
| B19 | ‘’ | 5UTR | 1 | 615 | 1 |
| B19 | ‘’ | 3UTR | 4970 | 5596 | 1 |
| B19 | ‘’ | PLA2 | 2990 | 3166 | 1 |
| BPV3 | AF406967 | Genome | 1 | 5276 | 1 |
| BPV3 | ‘’ | VP1 | 2219 | 5062 | 1 |
| BPV3 | ‘’ | Rep78 | 261 | 2216 | 1 |
| ChpPV | NC_038543 | Genome | 1 | 5205 | 1 |
| ChpPV | ‘’ | VP1 | 2619 | 5108 | 1 |
| ChpPV | ‘’ | Rep78 | 306 | 2441 | 1 |
| PmPV | NC_038542 | Genome | 1 | 5049 | 1 |
| PmPV | ‘’ | VP1 | 2307 | 4664 | 1 |
| PmPV | ‘’ | Rep78 | 296 | 2314 | 1 |
| RmPV | NC_038541 | Genome | 1 | 5342 | 1 |
| RmPV | ‘’ | VP1 | 2480 | 4942 | 1 |
| RmPV | ‘’ | Rep78 | 439 | 2490 | 1 |
| SePV | KF373759 | Genome | 1 | 4913 | 1 |
| SePV | ‘’ | VP1 | 2347 | 4878 | 1 |
| SePV | ‘’ | Rep78 | 319 | 2325 | 1 |
| SePV | ‘’ | ORF2 | 3119 | 3544 | 1 |
| SPV | NC_038540 | Genome | 1 | 4986 | 1 |
| SPV | ‘’ | VP1 | 2363 | 4819 | 1 |
| SPV | ‘’ | Rep78 | 307 | 2370 | 1 |
| SPV | ‘’ | ORF1 | 1667 | 2062 | 1 |
|  |  |  |  |  |  |
| **Artiparvovirus** |  |  |  |  |  |
| Aj_BtPV | NC_016752 | Genome | 1 | 4595 | 1 |
| Aj_BtPV | ‘’ | VP1 | 1965 | 4595 | 1 |
| Aj_BtPV | ‘’ | Rep78 | 84 | 1961 | 1 |
| Aj_BtPV | ‘’ | 5UTR | 1 | 83 | 1 |
|  |  |  |  |  |  |
| **Loriparvovirus** |  |  |  |  |  |
| SlPV | KP120516 | Genome | 1 | 4844 | 1 |
| SlPV | ‘’ | VP1 | 2187 | 4655 | 1 |
| SlPV | ‘’ | Rep78 | 146 | 1894 | 1 |
| SlPV | ‘’ | 5UTR | 1 | 145 | 1 |
| SlPV | ‘’ | 3UTR | 4656 | 4844 | 1 |
|  |  |  |  |  |  |
| **Tetraparvovirus** |  |  |  |  |  |
| PARV4 | NC_007018 | Genome | 1 | 5268 | 1 |
| PARV4 | ‘’ | VP1 | 2378 | 5122 | 1 |
| PARV4 | ‘’ | Rep78 | 283 | 2274 | 1 |
| PARV4 | ‘’ | 5UTR | 1 | 282 | 1 |
| PARV4 | ‘’ | 3UTR | 5121 | 5268 | 1 |
| BovineHoko1 | EU200669 | Genome | 1 | 5105 | 1 |
| BovineHoko1 | ‘’ | VP1 | 2274 | 5069 | 1 |
| BovineHoko1 | ‘’ | Rep78 | 202 | 2160 | 1 |
| ChimpPV4 | HQ113143 | Genome | 1 | 4971 | 1 |
| ChimpPV4 | ‘’ | VP1 | 2109 | 4853 | 1 |
| ChimpPV4 | ‘’ | Rep78 | 20 | 2011 | 1 |
| DeerTetraPV | NC_031670 | Genome | 1 | 5050 | 1 |
| DeerTetraPV | ‘’ | VP1 | 2171 | 4972 | 1 |
| DeerTetraPV | ‘’ | Rep78 | 77 | 2029 | 1 |
| DdPV | MG745671 | Genome | 1 | 5420 | 1 |
| DdPV | ‘’ | VP1 | 2336 | 5080 | 1 |
| DdPV | ‘’ | Rep78 | 306 | 2204 | 1 |
| EhBtPV1 | NC_016744 | Genome | 1 | 5065 | 1 |
| EhBtPV1 | ‘’ | VP1 | 2279 | 5038 | 1 |
| EhBtPV1 | ‘’ | Rep78 | 174 | 2252 | 1 |
| OvineHoko1 | JF504699 | Genome | 1 | 5249 | 1 |
| OvineHoko1 | ‘’ | VP1 | 2266 | 5067 | 1 |
| OvineHoko1 | ‘’ | Rep78 | 189 | 2159 | 1 |
| PorcineHoko1 | EU200677 | Genome | 1 | 5114 | 1 |
| PorcineHoko1 | ‘’ | VP1 | 2240 | 5017 | 1 |
| PorcineHoko1 | ‘’ | Rep78 | 176 | 2086 | 1 |
| RodentTetraPV | MG745669 | Genome | 1 | 5494 | 1 |
| RodentTetraPV | ‘’ | VP1 | 2395 | 5145 | 1 |
| RodentTetraPV | ‘’ | Rep78 | 308 | 2290 | 1 |
|  |  |  |  |  |  |
| **Protoparvovirus** |  |  |  |  |  |
| PorcinePV | D00623 | Genome | 1 | 2977 | 1 |
| PorcinePV | ‘’ | VP1 | 1188 | 2930 | 1 |
| PorcinePV | ‘’ | Rep78 | 3 | 764 | 1 |
| HamsterPV | U34255 | Genome | 1 | 4773 | 1 |
| CPV | NC_001539 | Genome | 1 | 5323 | 1 |
| CPV | ‘’ | VP1 | 2285 | 4540 | 1 |
| CPV | ‘’ | SAT | 272 | 2278 | 1 |
| CPV | ‘’ | Rep78 | 272 | 2278 | 1 |
| CPV | ‘’ | 5UTR | 1 | 130 | 1 |
| CPV | ‘’ | 3UTR | 3000 | 5323 | 1 |
| CPV | ‘’ | PLA2 | 2793 | 2999 | 1 |
| MVM | NC_001510 | Genome | 1 | 5149 | 1 |
| MVM | ‘’ | VP1 | 2286 | 2316 | 1 |
| MVM | ‘’ | VP1 | 2399 | 4557 | 2 |
| MVM | ‘’ | Rep78 | 114 | 2279 | 1 |
| Newlavirus | MZ813278 | Genome | 1 | 4772 | 1 |
| Newlavirus | ‘’ | VP2 | 2924 | 4696 | 1 |
| Newlavirus | ‘’ | VP1 | 1926 | 1953 | 1 |
| Newlavirus | ‘’ | VP1 | 2457 | 4696 | 2 |
| Newlavirus | ‘’ | SAT | 2931 | 3122 | 1 |
| Newlavirus | ‘’ | Rep78 | 68 | 1921 | 1 |
| OtterPV-1 | NC_030837 | Genome | 1 | 4639 | 1 |
| OtterPV-1 | ‘’ | VP1 | 2084 | 2111 | 1 |
| OtterPV-1 | ‘’ | VP1 | 2506 | 4628 | 2 |
| OtterPV-1 | ‘’ | Rep78 | 121 | 2097 | 1 |
| PPV | NC_001718 | Genome | 1 | 5075 | 1 |
| PPV | ‘’ | VP1 | 2287 | 2314 | 1 |
| PPV | ‘’ | VP1 | 2388 | 4549 | 2 |
| PPV | ‘’ | SAT | 2820 | 3026 | 1 |
| PPV | ‘’ | Rep78 | 292 | 2280 | 1 |
| Zsana | KT965075 | Genome | 1 | 4281 | 1 |
| Zsana | ‘’ | VP1 | 2668 | 4281 | 1 |
| Zsana | ‘’ | Rep78 | 1 | 1833 | 1 |
|  |  |  |  |  |  |
| **Icthamaparvovirus** |  |  |  |  |  |
| SynChPV | MN049932 | Genome | 1 | 4001 | 1 |
| SynChPV | ‘’ | VP1 | 2626 | 3720 | 1 |
| SynChPV | ‘’ | Rep78 | 216 | 2639 | 1 |
| SynChPV | ‘’ | ORF6 | 3177 | 3884 | 1 |
| SynChPV | ‘’ | ORF1 | 352 | 810 | 1 |
| SynChPV | ‘’ | NP | 1852 | 2562 | 1 |
| SynChPV | ‘’ | 5UTR | 1 | 215 | 1 |
| SynChPV | ‘’ | 3UTR | 3885 | 4001 | 1 |
|  |  |  |  |  |  |
| **Chaphamaparvovirus** |  |  |  |  |  |
| PPV7 | NC_040562 | Genome | 1 | 3999 | 1 |
| PPV7 | ‘’ | VP1 | 2546 | 3970 | 1 |
| PPV7 | ‘’ | Rep78 | 510 | 2528 | 1 |
| PPV7 | ‘’ | ORF1 | 196 | 597 | 1 |
| PPV7 | ‘’ | NP | 557 | 1306 | 1 |
| PPV7 | ‘’ | 5UTR | 1 | 195 | 1 |
| PPV7 | ‘’ | 3UTR | 3971 | 3999 | 1 |
| Cachavirus | MK448316 | Genome | 1 | 4226 | 1 |
| Cachavirus | ‘’ | VP1 | 2565 | 4118 | 1 |
| Cachavirus | ‘’ | Rep78 | 620 | 2611 | 1 |
| CChPV | MG846443 | Genome | 1 | 4228 | 1 |
| CChPV | ‘’ | VP1 | 2599 | 4227 | 1 |
| CChPV | ‘’ | Rep78 | 581 | 2602 | 1 |
| CKPV | MN265364 | Genome | 1 | 4427 | 1 |
| CKPV | ‘’ | VP1 | 2764 | 4254 | 1 |
| CKPV | ‘’ | Rep78 | 792 | 2771 | 1 |
| DrChPv | NC_032097 | Genome | 1 | 4284 | 1 |
| DrChPv | ‘’ | VP1 | 2651 | 4105 | 1 |
| DrChPv | ‘’ | Rep78 | 652 | 2658 | 1 |
| MkPV | MH670587 | Genome | 1 | 4442 | 1 |
| MkPV | ‘’ | VP1 | 2783 | 4273 | 1 |
| MkPV | ‘’ | Rep78 | 811 | 2790 | 1 |
| RChPV | KX272741 | Genome | 1 | 4222 | 1 |
| RChPV | ‘’ | VP1 | 2649 | 4067 | 1 |
| RChPV | ‘’ | Rep78 | 670 | 2634 | 1 |
| UrChPV | MN166196 | Genome | 1 | 3787 | 1 |
| UrChPV | ‘’ | VP1 | 2232 | 3701 | 1 |
| UrChPV | ‘’ | Rep78 | 260 | 2239 | 1 |
| BtlPV1 | MN996276 | Genome | 1 | 3411 | 1 |
| BtlPV1 | ‘’ | VP1 | 2059 | 3411 | 1 |
| BtlPV1 | ‘’ | Rep78 | 344 | 2101 | 1 |
| IcthPV | MN162688 | Genome | 1 | 3566 | 1 |
| IcthPV | ‘’ | VP1 | 2303 | 3565 | 1 |
| IcthPV | ‘’ | Rep78 | 269 | 2125 | 1 |
|  |  |  |  |  |  |

**Footnote: ^a^** Abbreviated virus name (see Table S1 for full name); **^b^** NCBI GenBank accession number; **^c^** Feature start position; **^d^** Feature end position; **^e^** Feature component index for discontiguous features (e.g., spliced genes).

**Abbreviations**: VP=capsid; ORF = open reading frame; UTR=untranslated region; IR=inverted repeated; AAP=assembly activating protein; NP=nucleoprotein; VL=variable loop.
