# Supplementary material for: Comparative analysis reveals the long-term coevolutionary history of parvoviruses and vertebrates
Source: PLoS Biol. 2022 Nov 29;20(11):e3001867. doi: 10.1371/journal.pbio.3001867 (PMC9707805; doi:10.1371/journal.pbio.3001867)
Supplement: S4 Table — (DOCX) [file pbio.3001867.s017.docx]

**Table S4.** Vertebrate endogenous parvoviral elements identified derived from dependoparvoviruses

| **Sequence ID ^a^** | **# Species^b^** | **Tax. rank^c^** | **Clade^d^** | **I^e^** | **E^f^** | **Upstream gene^g^** | **Downstream gene^h^** | **Age (Mya)^i^** | **Citation ^j^** |
| --- | --- | --- | --- | --- | --- | --- | --- | --- | --- |
|  |  |  |  |  |  |  |  |  |  |
| dependo.0-MacEug | 1 | Species | Oceania- |  |  | *NK* | *NK* |  | [1] |
| dependo.1-Whippomorpha | 24 | Clade | Lemuria- |  |  | PAX5 | PAX5 | 62 | [2] |
| dependo.2-Vespertilionidae | 9 | Clade | Neo- | NS |  | KIF6 | KIF6 |  | [2] |
| dependo.3-Lagomorpha | 3 | Order | Neo- | NS | Yes | NUP42 | GPNMB | 51 | [2] |
| dependo.4-Rhinocerotidae | 4 | Family | Lemuria- | VP |  | CCDC85A | LOC101927213 | 27.9 |  |
| dependo.5-Rhinocerotidae | 4 | Family | Lemuria- |  | Yes | USP38 | USP38 | 27.9 |  |
| dependo.6-Elephas | 1 | Genus | Neo- | NS | Yes | PIK3C3 | CELF4 |  | [3] |
| dependo.8-DasNov | 1 | Species |  |  |  | PRR14L | KCNH8 |  |  |
| dependo.9-Hyracoidea | 2 | Clade |  |  |  | *NK* | *NK* | 13.6 |  |
| dependo.10-Diprotodontia | 2 | Clade |  |  | Yes | TPD52 | TPD52 | 62.3 |  |
| dependo.11-Manis | 3 | Genus |  |  |  | *NK* | *NK* | 16.4 |  |
| dependo.12-Orycteropus | 1 | Species |  |  |  | *NK* | *NK* |  |  |
| dependo.13-Cercopithecidae | 3 | Family | Neo- | NS |  | HUNK | MIS18A | 19.4 |  |
| dependo.14-Cercopithecidae | 5 | Family | Neo- | NS | Yes | KRT6A | KRT6A | 19.4 |  |
| dependo.15-Colobus | 1 | Species | Neo- |  |  | ENSCAN | GAP43 | 19.4 |  |
| dependo.16-Canid | 5 | Family | Lemuria- |  |  | SPATS2L | MAIP1 |  |  |
| dependo.23-Camelidae | 5 | Family | Lemuria- |  |  | PNRC2 | RIDA | 11.7 |  |
| dependo.26-Strepsirrhini | 11 | Suborder | Lemuria- |  |  | ENSPSMG00000005325 | ACAD8 | 55 |  |
| dependo.27-Eulemur | 3 | Genus | Lemuria- | NS |  | *NK* | *NK* | 7.6 |  |
| dependo.28-DauMad | 1 | Species | Lemuria- |  |  | LRRK2 | CNTN1 |  |  |
| dependo.36-Phyllostomidae | 11 | Genus | Lemuria- |  |  | SMLR1 | TMEM200A | 37.5 |  |
| dependo.37-Rhinolophus | 2 | Genus | Lemuria- | NS |  | ENSRFEG00010017373 | CTTNBP2 | 27.6 |  |
| dependo.40-Phyllostomidae | 2 | Family |  |  |  | ENSRFEG00010011765 | FLRT2 |  |  |
| dependo.42-ChiLan | 1 | Species | Neo- | NS |  | *NK* | *NK* |  | [4] |
| dependo.43-OctDeg | 5 | Species | Neo- | NS | Yes | *NK* | *NK* |  | [4] |
| dependo.44-Bathyergidae | 2 | Family | Neo- |  |  | RPS12 | EYA4 | 34 |  |
| dependo.45-Bathyergidae | 2 | Family | Neo- |  |  | RPS12/SLC18B1 | EYA4 | 34 |  |
| dependo.46-Gliridae | 2 | Family | Neo- |  |  | *NK* | *NK* |  |  |
| dependo.47-PedCap | 1 | Species | Neo- |  |  | ENSCLAG00000029159 | TLE4 |  |  |
| dependo.48-Mus | *NK* | Genus | Neo- |  |  | *NK* | *NK* |  |  |
| dependo.49-ApoSyl | 1 | Species | Neo- |  |  | *NK* | *NK* |  |  |
| dependo.50-Muridae | *NK* | Family | Neo- |  |  | Pdzrn4 | Pdzrn4 | 26.5 |  |
| dependo.51-Muridae | 4 | Family | Neo- |  |  | Eif4b | ENSMSIG00000016524 | 26.5 |  |
| dependo.53-Cavia | *NK* | Genus | Neo- |  |  | *NK* | *NK* | 5.7 |  |
| dependo.54-Cavia | 3 | Genus | Neo- |  |  | RBFOX2 | Myo9-copy | 5.7 | [5] |
| dependo.55-Rodent | *NK* | Order | Neo- |  |  | ADNP | PARD6B |  |  |
| dependo.56-Cavia | 3 | Genus | Neo- |  |  | TRAT1 | MORC1 | 5.7 |  |
| dependo.58-CasCan | 1 | Species | Neo- |  |  | *NK* | *NK* |  |  |
| dependo.59-Hystricomorpha | 3 | Clade | Neo- |  |  | ENSODET00000026911 | MYH9 | 35 |  |
| dependo.60-MusAve | 1 | Species | Neo- |  |  | *NK* | *NK* |  |  |
| dependo.61-MusAve | 1 | Species | Neo- |  |  | *NK* | *NK* |  |  |
| dependo.62-MusAve | 1 | Species | Neo- |  |  | *NK* | *NK* |  |  |
| dependo.63-Muridae | NK | Family | Neo- |  | Yes | LRRGT00080 | LRRGT00080 |  |  |
| dependo.64-Muridae | NK | Family | Neo- |  |  | ENSHGL00100026153 | ENSHGL00100026154 |  |  |
| dependo.75-ApoSyl | 1 | Species | Neo- |  |  | *NK* | *NK* |  |  |
| dependo.76-Muridae | NK | Family | Neo- |  |  | Ces1d | Ces1d |  |  |
| dependo.86-Phyllostomidae | 10 | Family | Neo- |  |  | ENSRFEG00010019855 | HSF2 | 37.5 |  |
| dependo.87-MegLyr | 1 | Species | Neo- |  |  | ACVR1 | ACVR1C |  |  |
| dependo.88-MegLyr | 1 | Species | Neo- | NS |  | ADAMTS2 | U6 |  |  |
| dependo.92-CraTho | 1 | Species | Neo- |  | Yes | GRM7 | GRM7 |  |  |
| dependo.100-CunPac | 1 | Species | Neo- |  | Yes | MYH9 | MYH9 |  |  |
| dependo.160-GymLea | 1 | Species | Oceania- |  | Yes | ENSMODG00000048968 | ENSMODG00000048968 |  |  |
| dependo.162-MacEug | 1 | Species | Oceania- |  |  | *NK* | *NK* |  |  |
| dependo.163-MacEug | 1 | Species | Oceania- |  |  | *NK* | *NK* |  |  |
| dependo.164-MacEug | 1 | Species | Oceania- |  |  | *NK* | *NK* |  |  |
| dependo.174-PipPip | 1 | Species | Neo- | NS+VP |  | NAV3 | SYT1 |  |  |
| dependo.175-ProCap | 1 | Species | Neo- |  |  | *00NK* | *NK* |  |  |
| dependo.176-HetMeg | 1 | Species | Neo- |  |  | *NK* | *NK* |  |  |
| dependo.180-Rhinolophus | 2 | Genus | Neo- |  |  | ENSRFEG00010005236 | EPHA6 | 27.6 |  |
| dependo.187-GliGli | 1 | Species | Neo- |  |  | STPG2 | U6 |  |  |
| dependo.190-SarHar | 1 | Species | Oceania- |  |  | ZNF385D | SGO1 |  |  |
| dependo.191-SarHar | 1 | Species | Oceania- |  |  | ENSSHAG00000016096 | ENSSHAG00000017014 |  |  |
| dependo.192-SarHar | 1 | Species | Oceania- |  |  | ts | IGSF11 |  |  |
| dependo.197-OrnAna | 1 | Species | NK |  |  | ENSOANG00000038367 | ENSOANG00000041945 |  |  |
| dependo.199-OrnAna | 1 | Species | NK |  |  | TBC1D32 | ENSOANG00000014187 |  |  |
| dependo.201-ThaEle | 1 | Species | Shirdal- |  |  | ENSRFEG00010017356 | ENSRFEG00010017376 |  |  |
| dependo.202-ThaEle | 1 | Species | Shirdal- |  |  | ENSRFEG00010017356 | ENSRFEG00010017376 |  |  |
| dependo.203-GekJap | 1 | Species | Shirdal- |  |  | *NK* | *NK* |  |  |
| dependo.221-PelCas | 1 | Species | Shirdal- |  |  | ENSPCEG00000005322 | ENSPCEG00000005322 |  |  |
| dependo.365-Passeriformes | 7 | Order | Shirdal- |  |  | ANKRD1 | PCGF5 | 56 |  |
| dependo.366-Passeriformes | 20 | Order | Shirdal- |  |  | PTPN13 | AFF1 | 56 |  |
| dependo.367-Otididae | 1 | Family | Shirdal- |  |  | TMTC2 | SLC6A15 |  |  |
| dependo.369-GuaGua | 1 | Species | Shirdal- |  |  | *NK* | *NK* |  |  |
| dependo.370-PelCri | 1 | Species | Shirdal- |  |  | KLF6 | ENSSHBG00005014610 |  |  |
| dependo.371-EgrGar | 1 | Species | Shirdal- |  |  | PDE3A | AEBP2 |  |  |
| dependo.372-OpiHoa | 1 | Species | Shirdal- |  |  | CEP85L | SLC35F1 |  |  |
|  |  |  |  |  |  |  |  |  |  |

**Footnote: ^a^** Parvovirus-derived EVEs have been assigned standard IDs based on conventions established for endogenous retroviruses, wherein information about virus taxonomy and locus orthology are incorporated into the ID itself [6]. The ID comprises of three elements separated by hyphens. The first (i.e., leftmost) element is the classifier ‘endogenous parvoviral element’ (EPV). The second ID element comprises two subcomponents separated by a period – the first defines the taxonomic position of the EVE in relation to established *Flaviviridae* taxonomy, the second is a numeric ID that uniquely represents an EVE locus. The third ID component defines the known distribution of orthologous insertions among host species. If it is only known from a single species a shortened version of the Latin binomial species name is used. **^b^** Number of species in which this EPV locus was identified. **^c^** Taxonomic rank of species set in which EPV locus is found. **^d^** Subclade placement of EPV within this genus. **^e^** Names of intact ORFs found within any ortholog of this EPV set. **^f^** Evidence for expression of RNA from this EPV locus. **^g^** Nearest upstream gene **^h^** Nearest downstream gene ^i^ Minimum age of the locus as determined from orthology and species divergence dates obtained from the TimeTree database [7]. **^g^** Citation of paper where element published previously.

**Abbreviations**: MacEug=Macropus eugenii; DasNov=Dasypus novemcinctus; DauMad=Daubentonia madagascariensis; PedCap=Pedetes capensis; CasCan=Castor_canadensis; MusAve=Muscardinus_avellanarius; ApoSyl=Apodemus sylvaticus; MegLyr=Megaderma_lyra; CraTho=Craseonycteris thonglongyai; ChiLan=Chinchilla lanigera; CunPac=Cuniculus paca; GymLea=Gymnobelideus leadbeateri; PipPip=Pipistrellus pipistrellus; ProCap=Procavia capensis HetMeg=Heterohyrax brucei; GliGli=Glis glis; SarHar=Sarcophilus harrisii; OrnAna=Ornithorhynchus anatinus ThaEle=thamnophis elegans; GekJap=Gekko_japonicus; PelCas=Pelusios castaneus; PelCri=Pelecanus crispus; EgrGar=Egretta garzetta; GuaGua=Guaruba guarouba; OpiHoa=Opisthocomus hoazin; NS=replicase protein; VP=capsid protein.

**References**

1. Smith, R.H., et al., *Germline viral "fossils" guide in silico reconstruction of a mid-Cenozoic era marsupial adeno-associated virus.* Sci Rep, 2016. **6**: p. 28965.

2. Hildebrandt, E., et al., *Evolution of dependoparvoviruses across geological timescales – implications for design of AAV-based gene therapy vectors.* Virus Evolution, 2020.

3. Kobayashi, Y., et al., *An endogenous adeno-associated virus element in elephants.* Virus Res, 2018.

4. Arriagada, G. and R.J. Gifford, *Parvovirus-derived endogenous viral elements in two South American rodent genomes.* J Virol, 2014. **88**(20): p. 12158-62.

5. Valencia-Herrera, I., et al., *Molecular Properties and Evolutionary Origins of a Parvovirus-Derived Myosin Fusion Gene in Guinea Pigs.* J Virol, 2019. **93**(17).

6. Gifford, R.J., et al., *Nomenclature for endogenous retrovirus (ERV) loci.* Retrovirology, 2018. **15**(1): p. 59.

7. Kumar, S., et al., *TimeTree: A Resource for Timelines, Timetrees, and Divergence Times.* Mol Biol Evol, 2017. **34**(7): p. 1812-1819.
