# Supplementary material for: Comparative analysis reveals the long-term coevolutionary history of parvoviruses and vertebrates
Source: PLoS Biol. 2022 Nov 29;20(11):e3001867. doi: 10.1371/journal.pbio.3001867 (PMC9707805; doi:10.1371/journal.pbio.3001867)
Supplement: S5 Table — (DOCX) [file pbio.3001867.s018.docx]

**Table S5.** Vertebrate endogenous parvoviral elements identified derived from protoparvoviruses

| **Sequence ID ^a^** | **# Seq^c^** | **Tax. rank^d^** | **Clade^f^** | **I^g^** | **Upstream gene^i^** | **Downstream gene^j^** | **Age (Mya)^k^** | **Citation** |
| --- | --- | --- | --- | --- | --- | --- | --- | --- |
|  |  |  |  |  |  |  |  |  |
| proto.1-RatNor | 1 | Species | Neo- | VP | AABR07048228.1 | AABR07048231.1 |  | [1] |
| proto.2-MusSpr | 1 | Species | Neo- | NS/VP | PDZRN4 | PDZRN4 |  | [2] |
| proto.3-ApoSyl | 1 | Species | Neo- | NS/VP | GM14461 | UBE2E3 |  | [2] |
| proto.4-MusSpi | 1 | Species | Neo- | NS/VP | CCDC146 | CCDC146 |  |  |
| proto.5-GraMur | 1 | Species | Neo- |  | *NK* | *NK* |  |  |
| proto.102-TamTet | 1 | Species | Meso- | NS/VP | *NK* | *NK* |  |  |
| proto.219-TriMan | 1 | Species | Meso- |  | *NK* | *NK* |  |  |
| proto.101-MonDom | 1 | Species | Archeo- |  | U6 | ENSMODG00000006365 |  |  |
| proto.103-MonDom | 1 | Species | Archeo- |  | ENSMODG00000004341 | ENSMODG00000045240 |  |  |
| proto.104-MonDom | 1 | Species | Archeo- |  | U6 | ENSMODG00000006365 |  |  |
| proto.106-MyoCoy | 1 | Species | Archeo- |  | *NK* | *NK* |  |  |
| proto.107-MyoCoy | 1 | Species | Archeo- |  | *NK* | *NK* |  |  |
| proto.108-Hystricomorpha | 2 | Clade | Archeo- |  | *NK* | *NK* | 35 |  |
| proto.111-Sirenia | 2 | Order | Archeo- |  | *NK* | *NK* | 40 |  |
| proto.120-Cavia | 3 | Genus | Archeo- |  | NK | ENSCPOG00000022174 | 5.7 |  |
| proto.121-Cavia | 3 | Genus | Archeo- |  | *NK* | *NK* | 5.7 |  |
| proto.122-Cavia | 3 | Genus | Archeo- |  | ENSCPOG00000033273 | ENSCPOG00000026716 | 5.7 |  |
| proto.125-HydHyd | 1 | Species | Archeo- |  | *NK* | *NK* |  |  |
| proto.126-Octodontidae | 2 | Family | Archeo- |  | TRIML1 | FRG1 | 23.7 |  |
| proto.127-CapPil | 1 | Species | Archeo- |  | *NK* | *NK* |  |  |
| proto.128-CapPil | 1 | Species | Archeo- |  | *NK* | *NK* |  |  |
| proto.129-Dinomys | 1 | Species | Archeo- |  | MIPEP | AMER2 |  |  |
| proto.130-Dinomys | 1 | Species | Archeo- |  | OR4C6 | OR5AL1 |  |  |
| proto.131-CteSoc | 1 | Species | Archeo- |  | CDC73 | KCNT2 | 24.9 |  |
| proto.132-CteSoc | 1 | Species | Archeo- |  | COL24A1 | COL24A1 |  |  |
| proto.133-DolPat | 1 | Species | Archeo- |  | *NK* | *NK* |  |  |
| proto.134-DolPat | 1 | Species | Archeo- |  | *NK* | *NK* |  |  |
| proto.135-Octodontidae | 2 | Family | Archeo- |  | COL24A1 | COL24A1 | 23.7 |  |
| proto.136-OctMim | 1 | Species | Archeo- |  | *NK* | *NK* |  |  |
| proto.137-HydHyd | 1 | Species | Archeo- |  | *NK* | *NK* |  |  |
| proto.138-HydHyd | 1 | Species | Archeo- |  | *NK* | *NK* |  |  |
| proto.139-MyoCoy | 1 | Species | Archeo- |  | *NK* | *NK* |  |  |
| proto.140-MyoCoy | 1 | Species | Archeo- |  | *NK* | *NK* |  |  |
| proto.141-EreDor | 1 | Species | Archeo- |  | *NK* | *NK* |  |  |
| proto.142-CunPac | 1 | Species | Archeo- |  | *NK* | *NK* |  |  |
| proto.143-CunPac | 1 | Species | Archeo- |  | *NK* | *NK* |  |  |
| proto.144-MyoCoy | 1 | Species | Archeo- |  | *NK* | *NK* |  |  |
| proto.145-CteSoc | 1 | Species | Archeo- |  | *NK* | *NK* |  |  |
| proto.150-GymLea | 1 | Species | Archeo- |  | VPS13B | VPS13B |  |  |
| proto.151-Diprodontia | 1 | Clade | Archeo- |  | KLHL1 | KLHL1 | 48.9 |  |
| proto.152-SarHar | 1 | Species | Archeo- |  | ENSSHAG00000023532 | ENSSHAG00000027488 |  |  |
| proto.153-MacEug | 1 | Species | Archeo- |  | *NK* | *NK* |  |  |
| proto.154-PhaCin | 1 | Species | Archeo- |  | KCNJ6 | KCNJ6 |  |  |
| proto.155-GymLea | 1 | Species | Archeo- |  | ADGRL4 | ADGRL2 |  |  |
| proto.156-PhaCin | 1 | Species | Archeo- |  | *NK* | *NK* |  |  |
| proto.157-GymLea | 1 | Species | Archeo- |  | ENSPCIG00000007563 | ENSPCIG00000007563 |  |  |
| proto.158-MacEug | 1 | Species | Archeo- |  | ME3 | ME3 |  |  |
| proto.160-MacEug | 1 | Species | Archeo- |  | *NK* | *NK* |  |  |
| proto.161-MacEug | 1 | Species | Archeo- |  | CA10 | CA10 |  |  |
| proto.162-GymLea | 1 | Species | Archeo- |  | BACH2 | MAP3K |  |  |
| proto.163-PhaCin | 1 | Species | Archeo- |  | CA10 | CA10 |  |  |
| proto.164-MacEug | 1 | Species | Archeo- |  | AGPAT5 | XKR5 |  |  |
| proto.165-PhaCin | 1 | Species | Archeo- |  | U6 | ENSPCIG0000036639 |  |  |
| proto.166-MacEug | 1 | Species | Archeo- |  | *NK* | *NK* |  |  |
| proto.167-VomUrs | 1 | Species | Archeo- |  | MANCR | MANCR |  |  |
| proto.168-MyoCoy | 1 | Species | Archeo- |  | *NK* | *NK* |  |  |
| proto.169-MacEug | 1 | Species | Archeo- |  | *NK* | *NK* |  |  |
| proto.170-GraMur | 1 | Species | Archeo- |  | *NK* | *NK* |  |  |
| proto.171-MonDom | 1 | Species | Archeo- |  | ENSMODG00000007612 | ENSMODG00000047647 |  |  |
| proto.172-MonDom | 1 | Species | Archeo- |  | ENSMODG00000035635 | ENSMODG00000042352 |  |  |
| proto.173-GraMur | 1 | Species | Archeo- |  | *NK* | *NK* |  |  |
| proto.174-OctDeg | 1 | Species | Archeo- |  | PHF2 | BARX1 |  |  |
| proto.176-NanGal | 1 | Species | Archeo- |  | POT1-AS1 | POT1-AS1 |  |  |
| proto.177-EreDor | 1 | Species | Archeo- |  | *NK* | *NK* |  |  |
| proto.178-EreDor | 1 | Species | Archeo- |  | *NK* | *NK* |  |  |
| proto.179-MicTal | 1 | Species | Archeo- |  | *NK* | *NK* |  |  |
| proto.180-PhaCin | 1 | Species | Archeo- |  | ENPP2 | ENPP2 |  |  |
| proto.181-PhaCin | 1 | Species | Archeo- |  | KLF6 | ENSPCIG00000033363 |  |  |
| proto.182-PhaCin | 1 | Species | Archeo- |  | BTBD1 | BTBD1 |  |  |
| proto.183-PhaCin | 1 | Species | Archeo- |  | VPS8 | VPS8 |  |  |
| proto.184-PhaCin | 1 | Species | Archeo- |  | AGTPBP1 | AGTPBP1 |  |  |
| proto.185-PhaCin | 1 | Species | Archeo- |  | ENSPCIG00000032522 | ENSPCIG00000024782 |  |  |
| proto.186-PhaCin | 1 | Species | Archeo- |  | ENSPCIG00000015775 | ITGA6 |  |  |
| proto.187-PhaCin | 1 | Species | Archeo- |  | SFRP2 | ENSPCIG00000029650 |  |  |
| proto.188-PhaCin | 1 | Species | Archeo- |  | ENSPCIG00000031494 | ENSPCIG00000011842 |  |  |
| proto.189-Hystricomorpha | 2 | Clade | Archeo- |  | CES1C | CES1D | 35 |  |
| proto.190-EchTel | 1 | Species | Archeo- |  | GEMIN8P2 | *NK* |  |  |
| proto.191-MyoCoy | 1 | Species | Archeo- |  | *NK* | *NK* |  |  |
| proto.192-CteSoc | 1 | Species | Archeo- |  | *NK* | *NK* |  |  |
| proto.193-CteSoc | 1 | Species | Archeo- |  | CDC73 | KCNT2 |  |  |
| proto.194-SarHar | 1 | Species | Archeo- |  | ENSSHAG00000017845 | PCDH18 |  |  |
| proto.195-SarHar | 1 | Species | Archeo- |  | ENSSHAG00000026805 | ENSSHAG00000020835 |  |  |
| proto.196-SarHar | 1 | Species | Archeo- |  | ENSSHAG00000002243 | ENSSHAG00000028478 |  |  |
| proto.197-DinBra | 1 | Species | Archeo- |  | KSR1 | NOS2 |  |  |
| proto.198-MonDom | 1 | Species | Archeo- |  | ENSMODG00000036286 | ENSMODG00000036286 |  |  |
| proto.199-OryAfe | 1 | Species | Archeo- |  | TRNAG-CCC | CEMIP |  |  |
| proto.200-OryAfe | 1 | Species | Archeo- |  | LOC103197041 | LOC103197042 |  |  |
| proto.201-ChrAsi | 1 | Species | Archeo- |  | CCDC146 | CCDC146 |  |  |
| proto.202-ChrAsi | 1 | Species | Archeo- |  | CEMIP | CEMIP |  |  |
| proto.203-GymLea | 1 | Species | Archeo- |  | VPS8 | VPS8 |  |  |
| proto.204-GymLea | 1 | Species | Archeo- |  | EFHD1 | ITMC2 |  |  |
| proto.205-GymLea | 1 | Species | Archeo- |  | SFRP2 | ENSG00000280241 |  |  |
| proto.206-GymLea | 1 | Species | Archeo- |  | CA10 | CA10 |  |  |
| proto.207-GymLea | 1 | Species | Archeo- |  | BTBD1 | BTBD1 |  |  |
| proto.208-OctDeg | 1 | Species | Archeo- |  | HAS2 | CCDC115 |  |  |
| proto.209-MacEug | 1 | Species | Archeo- |  | VPS8 | VPS8 |  |  |
| proto.210-Diprotodontia | 1 | Order | Archeo- |  | OR13D1 | NIPSNAP3A | 62.3 |  |
| proto.211-MacEug | 1 | Species | Archeo- |  | *NK* | *NK* |  |  |
| proto.212-MacEug | 1 | Species | Archeo- |  | *NK* | *NK* |  |  |
| proto.213-MacEug | 1 | Species | Archeo- |  | *NK* | *NK* |  |  |
| proto.214-MacEug | 1 | Species | Archeo- |  | *NK* | *NK* |  |  |
| proto.215-MacEug | 1 | Species | Archeo- |  | ZPLD1 | ZPLD1 |  |  |
| proto.216-MacEug | 1 | Species | Archeo- |  | RPL7A | FRAT1 |  |  |
| proto.217-VomUrs | 1 | Species | Archeo- |  | ENSVURG00010005697 | NT5E |  |  |
| proto.218-VomUrs | 1 | Species | Archeo- |  | ENPP2 | ENPP2 |  |  |
|  |  |  |  |  |  |  |  |  |

**Footnote: ^a^** Parvovirus-derived EVEs have been assigned standard IDs based on conventions established for endogenous retroviruses, wherein information about virus taxonomy and locus orthology are incorporated into the ID itself [3]. The ID comprises of three elements separated by hyphens. The first (i.e., leftmost) element is the classifier ‘endogenous parvoviral element’ (EPV). The second ID element comprises two subcomponents separated by a period – the first defines the taxonomic position of the EVE in relation to established *Flaviviridae* taxonomy, the second is a numeric ID that uniquely represents an EVE locus. The third ID component defines the known distribution of orthologous insertions among host species. If it is only known from a single species a shortened version of the Latin binomial species name is used. **^b^** Number of species in which this EPV locus was identified. **^c^** Taxonomic rank of species set in which EPV locus is found. **^d^** Subclade placement of EPV within this genus. **^e^** Names of intact ORFs found within any ortholog of this EPV set. **^f^** Nearest upstream gene **^g^** Nearest downstream gene ^h^ Minimum age of the locus as determined from orthology and species divergence dates (obtained from TimeTree [4]).

**Abbreviations**: RatNor=Rattus norvegicus; MusSpr=Mus spretus; MusSpi=Mus spicelagus; TamTet=Tamandua tetradactyla; MonDom=Monodelphis domestica; CapPil=Capromys_pilorides; MacEug=Macropus eugenii; VomUrs=Vombatus ursinus; OctDeg=Octodon degus; OryAfe=Orycteropus afer; SarHar=Sarcophilus harrisii; MonDom=Monodelphis domestica; EchTel=Echinops telfairi; MyoCoy=Myocastor coypu; PhaCin=Phascolarctos cinereus; GymLea=Gymnobelideus leadbeateri; CteSoc=Ctenomys_sociabilis; EreDor=Erethizon dorsatum;ChrAsi=Chrysochloris asiatica; GraMur=Graphiurus_murinus; NanGal=Nannospalax_galili; CunPac=Cuniculus paca; DolPat=dolichotis patagonum; HydHyd=Hydrochoerus_hydrochaeris; NS=replicase protein; VP=capsid protein.

**References**

1. Kapoor, A., P. Simmonds, and W.I. Lipkin, *Discovery and characterization of mammalian endogenous parvoviruses.* J Virol, 2010. **84**(24): p. 12628-35.

2. Callaway, H.M., et al., *Examination and Reconstruction of Three Ancient Endogenous Parvovirus Capsid Protein Gene Remnants Found in Rodent Genomes.* J Virol, 2019. **93**(6).

3. Gifford, R.J., et al., *Nomenclature for endogenous retrovirus (ERV) loci.* Retrovirology, 2018. **15**(1): p. 59.

4. Kumar, S., et al., *TimeTree: A Resource for Timelines, Timetrees, and Divergence Times.* Mol Biol Evol, 2017. **34**(7): p. 1812-1819.
