# Supplementary material for: Comparative analysis reveals the long-term coevolutionary history of parvoviruses and vertebrates
Source: PLoS Biol. 2022 Nov 29;20(11):e3001867. doi: 10.1371/journal.pbio.3001867 (PMC9707805; doi:10.1371/journal.pbio.3001867)
Supplement: S6 Table — (DOCX) [file pbio.3001867.s019.docx]

**Table S6.** Vertebrate endogenous parvoviral elements that are not derived from proto- or dependoparvoviruses.

| **Sequence ID ^a^** | **# Species^b^** | **Tax. rank^c^** | **Clade^d^** | **Intact^e^** | **Upstream gene^f^** | **Downstream gene^g^** | **Age (Mya)^h^** |  | **Citation^i^** |
| --- | --- | --- | --- | --- | --- | --- | --- | --- | --- |
|  |  |  |  |  |  |  |  |  |  |
| **Amdoparvovirus** |  |  |  |  |  |  |  |  |  |
| amdo.1-EllLut | 1 | Species | Mammal | NS | NK | NK |  |  | [1] |
| amdo.2-EllLut | 1 | Species | Mammal | NS-MAFG | MAFG | MAFG |  |  | [1] |
| ap.101-Serpentes-UR | 4 | Suborder | Serpentes | NS/VP | R3HDM2 | INHBC | 55.2 |  | [1] |
| ap.102-Hyracoidae | 2 | Family | Mammal |  | PVT1 | CCDC26 | 10.2 |  | [1] |
| ap.103-OryAfe | 1 | Orycteropus | Mammal |  | *NK* | *NK* |  |  | [1] |
| ap.104-SarHar | 1 | Species | Mammal |  | *NK* | *NK* |  |  | [1] |
| ap.105-PtyMuc | 1 | Species | Serpentes |  | GRM5 | GRM5 |  |  |  |
|  |  |  |  |  |  |  |  |  |  |
| **Erythroparvovirus** |  |  |  |  |  |  |  |  |  |
| erythro.1-IndInd | 1 | Species |  | NS/VP | IL1RAPL1 | IL1RAPL1 |  |  |  |
| erythro.2-DolPat | 1 | Species |  |  | *NK* | ENSODEG00000000416 |  |  |  |
|  |  |  |  |  |  |  |  |  |  |
| **Unclassified *Parvoviridae*** |  |  |  |  |  |  |  |  |  |
| parvovirinae.1-ScyCan | 1 | Species |  |  | *NK* | *NK* |  |  |  |
| parvovirinae.2-TetNig | 1 | Species |  |  | *NK* | *NK* |  |  |  |
| parvovirinae.7-BraVar | 1 | Species |  |  | *NK* | *NK* |  |  |  |
| parvovirinae.20-Laurasiatheria | 13 | Superorder |  |  | GGT5 | GGT5 | 86.3 |  |  |
| parvovirinae.22-Laurasiatheria | 76 | Superorder |  |  | PCDH19 | PCDH19 | 86.3 |  |  |
| parvovirinae.23-Supraprimates |  | Superorder |  |  | Limbin | Limbin | 87.2 |  | [2] |
|  |  |  |  |  |  |  |  |  |  |
| **Ichthamaparvovirus** |  |  |  |  |  |  |  |  |  |
| ichthama.1-Hippocampus | 1 | Species |  |  | ENSHCOG00000009382 | *NK* |  |  | [3] |
| ichthama.2-Serpentes-UR | 6 | Unranked |  | NS/ ORF2 | BHLHE23 | YTHDF1 | 55.2 |  |  |
|  |  |  |  |  |  |  |  |  |  |
|  |  |  |  |  |  |  |  |  |  |

**Footnote: ^a^** Parvovirus-derived EVEs have been assigned standard IDs based on conventions established for endogenous retroviruses, wherein information about virus taxonomy and locus orthology are incorporated into the ID itself [4]. The ID comprises of three elements separated by hyphens. The first (i.e., leftmost) element is the classifier ‘endogenous parvoviral element’ (EPV). The second ID element comprises two subcomponents separated by a period – the first defines the taxonomic position of the EVE in relation to established *Flaviviridae* taxonomy, the second is a numeric ID that uniquely represents an EVE locus. The third ID component defines the known distribution of orthologous insertions among host species. If it is only known from a single species a shortened version of the Latin binomial species name is used. **^b^** Number of species in which this EPV locus was identified. **^c^** Taxonomic rank of species set in which EPV locus is found. **^d^** Subclade placement of EPV within this genus. **^e^** Names of intact ORFs found within any ortholog of this EPV set. **^f^** Nearest upstream gene **^g^** Nearest downstream gene ^h^ Minimum age of the locus as determined from orthology and species divergence dates obtained from the TimeTree database [5]. **^g^** Citation of paper where element published previously.

**Abbreviations**: EllLut=Ellobius lutescens; OryAfe=Orycteropus afer; SarHar=Sarcophilus harrisii; PtyMuc=Ptyas mucosa; IndInd=Indri indri; DolPat=dolichotis patagonum; ScyCan=Scyliorhinus canicular TetNig=Tetraodon nigroviridis; BraVar=Bradypus variegatus. NS=replicase protein; VP=capsid protein; ORF=open reading frame.

**References**

1. Pénzes, J.J., et al., *Endogenous amdoparvovirus-related elements reveal insights into the biology and evolution of vertebrate parvoviruses.* Virus evolution, 2018. **4**(2): p. vey026-vey026.

2. Liu, H., et al., *Widespread endogenization of densoviruses and parvoviruses in animal and human genomes.* J Virol, 2011. **85**(19): p. 9863-76.

3. Pénzes, J.J., et al., *An Ancient Lineage of Highly Divergent Parvoviruses Infects both Vertebrate and Invertebrate Hosts.* Viruses, 2019. **11**(6).

4. Gifford, R.J., et al., *Nomenclature for endogenous retrovirus (ERV) loci.* Retrovirology, 2018. **15**(1): p. 59.

5. Kumar, S., et al., *TimeTree: A Resource for Timelines, Timetrees, and Divergence Times.* Mol Biol Evol, 2017. **34**(7): p. 1812-1819.
