# Supplementary material for: Comparative analysis reveals the long-term coevolutionary history of parvoviruses and vertebrates
Source: PLoS Biol. 2022 Nov 29;20(11):e3001867. doi: 10.1371/journal.pbio.3001867 (PMC9707805; doi:10.1371/journal.pbio.3001867)
Supplement: Attachment — Submitted filename: Response.docx [file pbio.3001867.s020.docx]

**REVIEWERS' COMMENTS:**

**Reviewer #1:**

*Campbell et al. have employed multiple methods to map the diversity of endogenous parvovirus sequences in vertebrate genomes. They both expand the known diversity of these ssDNA viruses, while further dating their divergence with vertebrate evolution. Through these findings, the authors provide an evidence-based narrative for host adaptation and provide insight into host-virus coevolutionary dynamics. In particular, parvovirus phylogenies track with mammalian vicariance. The authors further provide evidence for much more extensive rodent parvovirus clades, that seem to include recombination events of VP/Cap genes - this seems like a readied reservoir of dispersal and mammalian host-switching as to give parvo success in becoming more host-ubiquitous. They have further collated their data into an open, cross-platform database (GLUE) that should be a standard of the field.*

*The systematic review of endogenous viral elements (EVEs) in existing genome data to reconstruct virus evolution history is entwined in an understanding of the host evolution as well. Beyond retroviral elements, identifying EVEs of other viral classes due to rare germline incorporation events seems critical in understanding the virus-host co-evolution taking place under varied transmission regimes through time.*

Thank you for these positive and encouraging comments.

***I have a few comments and need for clarification from the authors:***

*Substitution models. The authors utilized GTR to construct a phylogeny of EPV ORF fragments. This makes sense in clocking variation post-genome incorporation (even while the authors acknowledge in Discussion the unknown of selection/drift on their maintenance). Do the authors see any framework under which host-specific substitution rates (often driven by selection, particularly during host shifts) could make determining the divergence of Parvo ORFs imprecise? Perhaps the large temporal scale here and match to vicariance is just too parsimonious… but I do wonder about the role of rodent host-shifts and recombinations also creating non-clock temporal abnormalities in the substitution rate that could make incorporation events primed for poor dating*.

We agree with the reviewer that divergence among EPV sequences reflects different kinds of substitution pressures and is an unreliable source of temporal signal - not only because we cannot easily untangle the impact of purifying selection in the virus versus the host (some EPVs retain ORFs suggesting exaptation and host-level purifying selection), but also because of host-specific (or locus-specific) differences in the neutral substitution rate. We therefore only used EPV sequences to infer topological relationships within the parvovirus phylogeny and deliberately avoided the use of molecular clock-based calibrations. We restricted our phylogenetic inferences with respect to EPVs to topology only. For dating we relied on orthology and host divergence calibrations (which are informed by the vertebrate fossil record). We now make this more explicit in our introduction (p4 lines 9-13).

*Relative frequency in mammalian genomes (Results, page 7, Table 2). Can you expand on this and/or speak to the significance? Given the paucity of genomes to capture full vertebrate diversity, and the relative over-representation of mammalian species relative to their 'weight' in biodiversity… do the authors believe this mammalian bias would hold? Are we missing a number of events in non-mammalian vertebrates that could shift your conclusion?*

In our revised manuscript we have included a statistical test of the biased distribution (p6: lines 18-24).

*Page 7, line 1: *greater or less* than RT DNA viruses? A word seems to be missing.*

Thank you, we have fixed this error.

**Reviewer #2:**

*This manuscript by Campbell and colleagues is a comprehensive examination on the long-term evolution of parvoviruses. This study is complex but interesting and compelling as the authors catalogue EPV sequences from 752 vertebrate genomes and they do so in a manner which is highly transparent and reproducible in the form of an open database. GLUE is a computational framework that not captures sequence data and associated metadata but also allows for genomic analysis to be performed in an efficient, standardised and reproducible manner. The software itself is versatile as demonstrated by its utility to a wide range of different viruses such as SARS-CoV-2, HIV and HCV. In essence the platform to support this type of analysis is excellent. However, with that said I think that the framework and its application has been published for other viruses in the past so at times it seems that this manuscript is sometimes too focused on the novelty of Parvovirus-GLUE when the authors could*

*have easily found over 300 EPV sequences without this framework.*

*Overall, the manuscript is quite dense and there is a lot to unpack and while the authors attempt to navigate the reader through the study I do think that clarity is needed in places as some sections could easily be reduced or even completely removed. For example, one could easily assume that novel exogeneous virus species may be identified when doing such large data screening approaches but in the absence of other experimental support they are purely in silico findings and should be treated with caution. Also do such findings really add to the study?*

We appreciate these constructive remarks. In our revised draft we have simplified the presentation of our findings to create a more streamlined manuscript which we think reads much more clearly. Following the reviewer’s suggestion, we removed the section on putatively exogenous parvoviruses identified in our study. We have also simplified and streamlined our introduction and results. Our revised draft is more squarely focussed on the EPVs reported in our study and their implications for parvovirus evolution.

**Other comments:**

*1. Is there a minimum sequence threshold for an endogenous parvoviral element as I am thinking the phylogenetic signal with smaller fragments may be quite low so what quality control steps are there.*

The threshold of detection was empirically determined to firmly exclude false positive hits by comparing all putative EPV sequences identified in our screen to all previously published sequences in NCBI GenBank’s non-redundant (nr) nucleotide sequence database. Sequences that were not unambiguously parvoviral – i.e., they matched to parvoviral sequences much more closely than they did to any other virus or host sequence. There is a ‘grey zone’ of remote similarity where it is difficult to discriminate between false positives and real EPVs that are just very old and divergent. In our study we conservatively set thresholds to exclude any sequences that were not unambiguously derived from parvoviruses - hence our initial screen did not detect the oldest EPV locus (epv-23-supraprimates – reported previously) which is relatively short and highly mutated.

*2. Page 8 Line 11: "Fig.3a" and "Fig.3b) don't exist. Is this supposed to be Figure 3?*

Thank you we have fixed this error

*3. Can the authors be more explicit in how time-calibrated vertebrate phylogenies were performed. The text merely states TimeTree which I presume the authors favoured over Bayesian methods such as BEAST due to its speed?*

We apologise for not explaining this more clearly. We did not use molecular clock-based dating in our paper – as discussed in the response to reviewer 1, this is not feasible because it is not possible to discriminate within-host evolutionary change of EVE sequences from evolution that occurred in viruses prior to integration. Therefore, we relied on minimum ages inferred from the presence of orthologous insertions in related species. The presence of an ortholog indicates that integration occurred before species divergence, TimeTree^TM^ is a comprehensive database of organism divergence date estimates compiled from published literature. These estimates sometimes incorporate the use of molecular data, but importantly they also draw on the fossil record, an orthogonal data source. In our revised manuscript we include outline this approach to data more explicitly (p4, lines 9-13, and p21, line 7).

*4. What about the limitations of sampling as I am sure it heavily biased towards certain areas and I wonder how this limits modelling the ancestral biogeographical range of protoparvovirus hosts. The authors should make reference to the limitations of this study such that it is really based on opportunistic sampling available in GenBank.*

In our revised draft we comment on the limitations of our study, which we agree relies on opportunistic sampling (p15, lines 5-9).

*5. Will the Parvovirus-GLUE project be updated and maintained in the future?*

In our revised draft we explicitly discuss how the Parvovirus-GLUE project can be updated and maintained in the future (p6, lines 1-8).
