# Supplementary material for: Comparative analysis reveals the long-term coevolutionary history of parvoviruses and vertebrates
Source: PLoS Biol. 2022 Nov 29;20(11):e3001867. doi: 10.1371/journal.pbio.3001867 (PMC9707805; doi:10.1371/journal.pbio.3001867)
Supplement: Attachment — Submitted filename: Response.docx [file pbio.3001867.s021.docx]

**REVIEWERS' COMMENTS:**

**1) The legend to Figure 4 legend more explicitly say where the mammalian phylogeny comes from. This is said somewhere in the methods, but it is hard to find it, and it would not hurt to have this info also in the figure legend.**

*Figure 4 shows a phylogeny of subfamily Parvovirinae. The legend explains how the phylogeny was constructed as follows:*

*‘The complete phylogeny, which is midpoint rooted for display purposes, was reconstructed using a multiple sequence alignment spanning 270 amino acid residues in the Parvovirus Rep protein and the LG likelihood substitution model.’*

*We think perhaps the reviewer is referring to figure 3 - accordingly we have added to the legend that this phylogeny was obtained from TimeTree, as referenced in our previous response. We also now state this in the legends to Figures 5c and 5d, which show time-scaled mammalian phylogenies.*

**2) In a previous version of the manuscript, I think that the phylogenetic analysis used to have a section of its own in the main text. In my opinion it was helpful for the reader to find this information in just one place. Now this seems to be scattered around different methodological sections in the main text and some bits seem to have been lost (e.g. I could not find the reference for the GTR model of sequence evolution in the main text). I am not sure if this was done to follow the reviewer’s suggestions, but I do have to say that I like to have a place where I can go and quickly find the general overview about the phylogenetic analysis. In fact, it might be worth adding even more information on the phylogenetic analysis as it had before (apart from table 3). Given the limited time I had to read the latest version, I might have missed this in my last reading, if so sorry. On the other hand, having all this information in one place could have helped me (and others) to quickly find all the info on phylogenetic analysis.**

*In our revised manuscript we have rearranged the text so that the phylogenetic information is all in one place. This did not entail a major change only some restructuring. We agree this information is more easily accessed this way.*

*Note - The GTR model of nucleotide substitution was used only to reconstruct the phylogenetic relationships of EPV orthologs, which we used to help confirm orthology.*

**3) I think the authors could more explicitly say (simply add this information in the text) if the phylogeographic analysis was done solely in the maximum likelihood tree (that was my impression) and perhaps add some short discussion on how the support of the nodes might affect their inferences.**

*We have made the proposed changes to the text (p12 – lines 9-26). In addition, we have removed all reference to an analysis in which we modelled the ancestral biogeographical range of protoparvovirus hosts, because we think that it was adding relatively little and creating some confusion. With this analysis removed, we think it is now clear that our biogeographic inferences are based solely on the maximum-likelihood phylogeny of protoparvovirus and EPV sequences shown in figure 5a. We emphasise in the text that the internal nodes in this phylogeny have robust support, and the topology is very unlikely to be an artefact of our analysis.*
